# A Further Theoretical Study of Capacitive Pressure Sensors Based on Thin Film Elastic Deflection and Parallel Plate Capacitor: Refined Closed-Form Solution and Numerical Calibration

**DOI:** 10.3390/s22082848

**Published:** 2022-04-07

**Authors:** Ying Guo, Bo Li, Qi Zhang, Xiao-Ting He, Jun-Yi Sun

**Affiliations:** 1 School of Civil Engineering, Chongqing University, Chongqing 400045, China; guoying@cqu.edu.cn (Y.G.); 202116131339@cqu.edu.cn (B.L.); 202016021045@cqu.edu.cn (Q.Z.); hexiaoting@cqu.edu.cn (X.-T.H.); 2 Key Laboratory of New Technology for Construction of Cities in Mountain Area (Chongqing University), Ministry of Education, Chongqing 400045, China

**Keywords:** capacitive pressure sensor, parallel plate capacitor, elastic deflection, annular membrane, closed-form solution

## Abstract

The capacitive pressure sensor based on thin film elastic deflection and a parallel plate capacitor uses a non-conductive elastic annular thin film centrally connected to a conductive, rigid, flat, concentric-circular thin plate as a pressure sensing unit. On application of pressure, the non-conductive thin film deflects elastically, which in turn moves the conductive thin plate (as a movable upper electrode plate of the parallel plate capacitor) towards the lower electrode plate, resulting in a change in the capacitance of the capacitor. Therefore, the applied pressure can be determined by measuring the capacitance change, based on the closed-form solution for the elastic behavior of the annular thin film under pressure. Such capacitive pressure sensors are more suitable for large-sized sensors such as those used for building-facade wind pressure measurements, etc. In this paper, a further theoretical study of such capacitive pressure sensors is presented. The newly presented, more refined closed-form solution can greatly reduce the output pressure error under the same input capacitance, in comparison with the previously presented closed-form solution. A numerical example of how to use the resulting closed-form solution to numerically calibrate input–output characteristics is given for the first time. The variation trend of pressure operation ranges and input–output characteristics with important parametric variations, which can be used for guiding the design of such capacitive pressure sensors, is investigated.

## 1. Introduction

Many thin films are capable of exhibiting large elastic deflection under transverse loading [1,2,3,4,5,6], which provides the possibility of the design and development of elastic-deflection-based devices [7,8,9,10,11,12,13,14]. Pressure sensors based on thin film elastic deflection have widespread applications in many areas, such as bio-medical applications, robotics, automobiles, and environmental monitoring, and capacitive structures are widely used. These capacitive types of pressure sensors convert the elastic deformation of thin films, corresponding to the pressure applied on the thin films, into a change in capacitance. They usually use single-crystal silicon and polysilicon [15], polymer/ceramic [16], low-temperature co-fired ceramic [17], silicon carbide [18,19], or graphene–polymer heterostructure [20,21,22,23] thin films in microelectromechanical systems (MEMS), and have the advantages of low cost, small volume, high stability, high sensitivity, low temperature drift, and lower sensitivity to environment effects.

**Figure 1:**
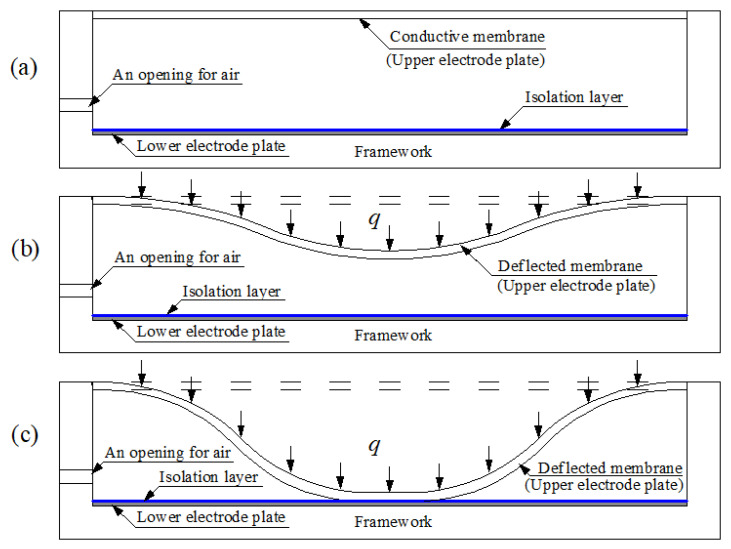
Sketch of the structure and modes of operation of a traditional capacitive pressure sensor: (**a**) the status without application of the pressure *q*; (**b**) non-touch mode of operation under the pressure *q*; (**c**) touch mode of operation under the pressure *q*.

Figure 1 illustrates the typical structure and modes of operation of a traditional capacitive pressure sensor. On application of a pressure *q*, the conductive membrane, as the upper electrode plate of the capacitor, elastically deflects in response to the applied pressure *q*. This elastic deflection is a measure of the applied pressure *q* and also changes the capacitance of the capacitor. Therefore, the applied pressure *q* can be determined by measuring the change in capacitance. In the so-called normal mode of operation, the deflected conductive membrane, as the upper electrode plate of the capacitor, is always kept at a distance away from the isolation layer coating the lower electrode plate, as shown in Figure 1b, and thus a device operating in this state may also be called a non-touch-mode capacitive pressure sensor. When in the so-called touch mode of operation, the deflected conductive membrane is always kept in contact with the isolation layer, as shown in Figure 1c, and therefore a device operating in this state is called a touch-mode capacitive pressure sensor. Usually, the output capacitance of a non-touch-mode capacitive pressure sensor is nonlinear with respect to the input pressure changes, and the sensitivity in the near-linear region is not high enough to ignore the many stray capacitance effects. Touch-mode capacitive pressure sensors are known to have robust structures to withstand harsh industrial environments and higher sensitivity by one or two orders of magnitude than in the normal mode of operation in the near-linear operation range, so that some of the stray capacitance effects can be neglected. Moreover, the output capacitance of touch-mode devices is mainly the isolation-gap capacitance of the touched area due to the very thin isolation layer, and its capacitance per unit area is much larger than the air-gap capacitance in the untouched area. This is the main reason why touch-mode capacitive pressure sensors are often called linear sensors: the change in the touched area is usually designed to be almost proportional to the applied pressure *q*, and thus the output capacitance–input pressure characteristic is nearly linear.

However, there are two types of difficulties or problems in the design of traditional capacitive pressure sensors. Firstly, there are difficulties in the preparation or selection of conductive elastic membranes. The conductive membrane is used as both the movable upper electrode plate of the capacitor and the deformation element that elastically responds to the applied pressure. Therefore, the preparation or selection of the conductive membrane depends not only on its high electrical conductivity but also on its good elastic deformation ability, which obviously places high requirements on the preparation or selection of materials. Secondly, there are difficulties in balancing the linear input–output characteristic and the wide operational pressure range. It is in fact very difficult to design a touch-mode capacitive pressure sensor with a wide operational pressure range and a nearly linear characteristic between the output capacitance and the input pressure. In general, when the operational pressure range is too wide, there often is a strong nonlinear relationship between the change in touched area and the applied pressure. As a result, the designer must often choose between a wide operational pressure range and a nearly linear input–output characteristic.

In our earlier work [24], we proposed an improved capacitive pressure sensor using non-conductive elastic membranes and conductive rigid thin plates instead of traditional conductive elastic membranes. The topside structure of the proposed capacitive pressure sensor uses a non-conductive elastic annular membrane (as an elastic deformation element to respond to the applied pressure), whose inner edge is rigidly connected to the outer edge of a conductive, rigid, flat, concentric-circular thin plate (as a movable upper electrode plate of the capacitor), to substitute for the dual-function topside structure of traditional capacitive pressure sensors, i.e., the traditional conductive elastic membrane used as both an elastic deformation element and an upper electrode plate. The proposed capacitive pressure sensor uses independent elastic deformation elements and upper electrode plates, overcoming the shortcomings of traditional capacitive pressure sensors. Non-conductive membranes with very good elasticity are abundant, and rigid thin plates with high electrical conductivity are easier to find, making the preparation or selection of materials very easy. Furthermore, the convenience of material preparation or selection allows a wider range of material parameters to be selected, such as the Poisson’s ratio, Young’s modulus of elasticity, and the thickness of the membrane, as well as the radius of the conductive, rigid, flat, concentric-circular thin plate. In particular, the conductive, rigid, flat, concentric-circular thin plate, as a movable upper electrode plate, forms a parallel plate capacitor with the flat lower electrode plate, and the calculation for a parallel plate capacitor is well known to be easier than that for a non-parallel plate capacitor. All of these advantages provide great convenience for balancing a wide operational pressure range and a nearly linear input–output characteristic.

The improved capacitive pressure sensors proposed in [24] have many advantages over traditional capacitive pressure sensors: they are more suitable for large-volume (size) sensors such as those used for building-facade wind pressure measurements, etc. However, our earlier work [24] failed to accurately solve the behavior of the elastic deformation of the annular membrane analytically, due to the complexity of the problem. An accurate analytical solution is usually very important for sensor design, and the closed-form solution presented in [24] could not meet the design requirements of the proposed capacitive pressure sensor, due to the adopted assumption condition that the rotation angle *θ* of the annular membrane is so small that “sin*θ* = tan*θ*” can be used to replace “sin*θ* = 1/(1 + 1/tan^2^*θ*)^1/2^”. Obviously, such an assumption inevitably introduces computational errors and affects the accuracy of the closed-form solution presented in [24] when the rotation angle of the annular membrane is relatively large, i.e., when the applied pressure is relatively large. As is well known, the sine function sin*θ* can be approximated by the tangent function tan*θ* only when *θ* is relatively small, and a large rotation angle *θ* will give rise to a significant approximation error. For instance, the error caused by approximating sin*θ* to tan*θ* is about 1.54% when *θ* = 10°, 6.42% when *θ* = 20°, 15.47% when *θ* = 30°, and 30.54% when *θ* = 40°. In fact, the rotation angle *θ* of the annular membrane in the proposed parallel-plate-capacitor-based pressure sensor may exceed 40°. Therefore, it is necessary to reject the approximation of replacing “sin*θ* = 1/(1 + 1/tan^2^*θ*)^1/2^” with “sin*θ* = tan*θ*” in the derivation of the closed-form solution. Hence, the behavior of the elastic deformation of the annular membrane under pressure must be analytically solved again.

Our earlier work [24] also failed to give an illustration of how to use the closed-form solution to achieve the numerical calibration of the relationship between the output pressure and the input capacitance of the proposed capacitive pressure sensor. As can be seen below, changes in material parameters such as the initial gap between the upper and lower electrode plates of the parallel plate capacitor, the Young’s modulus of elasticity, and the thickness of the membranes, have an important effect on the relationship between the output pressure and the input capacitance of the proposed capacitive pressure sensor. Therefore, the numerical calibration plays a very important role in the design phase of the proposed capacitive pressure sensor as it determines the required operational pressure ranges and input–output characteristics. In other words, it is impossible to achieve the required operational pressure ranges and input–output characteristics by just changing the actual materials used (i.e., by experimental calibration). Thus, closed-form solutions are of unparalleled and irreplaceable value in sensor design. Therefore, as a purely theoretical study, since the closed-form solution is given, it is necessary to address how to use the closed-form solution to achieve the numerical calibration of the proposed capacitive pressure sensor. However, our previous work [24] failed to do this.

This paper renames the improved capacitive pressure sensor proposed in [24] as a capacitive pressure sensor based on thin film elastic deflection and a parallel plate capacitor (or an elastic-deflection-and-parallel-plate-capacitor-based pressure sensor for short) and presents a further theoretical study of the pressure sensor. In this paper, in order to improve the accuracy of analytical solutions, the assumption adopted in [24] is rejected, resulting in a new and more refined closed-form solution for the behavior of the elastic deformation of the annular membrane. For the first time, examples are given to illustrate how to use the resulting closed-form solution to achieve the numerical calibration of the relationship between the output pressure and input capacitance of the elastic-deflection-and-parallel-plate-capacitor-based pressure sensor. In addition, the effect of important parametric variations on the input–output characteristics is also discussed numerically. The novelty or innovation of this paper mainly lies in the following three aspects. A new and more refined closed-form solution is presented, where the newly presented closed-form solution can greatly reduce the pressure measurement error for the same input capacitance (i.e., under the same maximum elastic deflection, in comparison with the previously presented closed-form solution. A numerical example of how to use the resulting closed-form solution to numerically calibrate the input–output characteristics is given for the first time. The effect of important parametric variations on the input–output characteristics is addressed, which has important theoretical significance for guiding the design of capacitive pressure sensors based on thin film elastic deflection and a parallel plate capacitor. By changing some important parameters and carrying out a series of numerical calibrations, the variation trend of the operational pressure ranges and input–output characteristics with important parametric variations can be found. This can clarify how to appropriately prepare or select materials to achieve the desired operational pressure ranges and input–output characteristics.

The paper is organized as follows. In the following section, the structure, the mode of operation, and the working principle of the elastic-deflection-and-parallel-plate-capacitor-based pressure sensor are briefly described. In Section 3, the behavior of the elastic deformation of the annular membrane of the elastic-deflection-and-parallel-plate-capacitor-based pressure sensor under pressure is analytically solved again, the assumption condition adopted in [24] is rejected, and a new and more refined closed-form solution is given. In Section 4, some important issues are addressed. The validity of the closed-form solution obtained in Section 3 is first addressed. Secondly, the new closed-form solution given in this paper is numerically compared with the one given in [24], in terms of the pressure measurement error under the same maximum elastic deflection (i.e., under the same input capacitance). Next, an example is given to illustrate how to use the closed-form solution obtained in Section 3 to achieve the numerical calibration of the relationship between the output pressure and input capacitance of the elastic-deflection-and parallel-plate-capacitor-based pressure sensor. Finally, the effect of important parametric variations on input–output relationships is numerically discussed, showing how the required operational pressure ranges and input–output characteristics can be achieved based on a series of numerical calibrations. Concluding remarks are given in Section 5.

## 2. Materials and Methods

**Figure 2:**
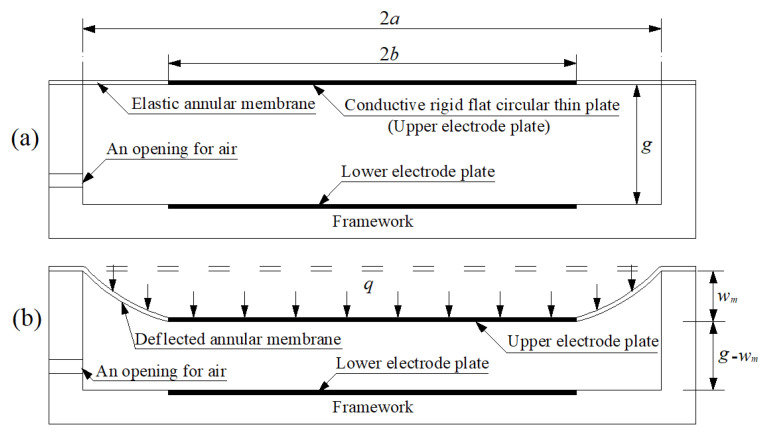
Sketch of an elastic-deflection-and-parallel-plate-capacitor-based pressure sensor: (**a**) without application of pressure; (**b**) with applied pressure *q*.

The structure or geometry of the proposed elastic-deflection-and-parallel-plate-capacitor-based pressure sensor is shown in Figure 2a, where *a* denotes the outer radius of the initially flat non-conductive elastic annular membrane, *b* denotes the inner radius of the annular membrane, as well as the outer radius of the conductive, rigid, flat, concentric-circular thin plate, and *g* denotes the initial gap between the initially flat non-conductive elastic annular membrane and the flat lower electrode plate. The inner edge of the initially flat non-conductive elastic annular membrane is rigidly connected to the outer edge of the conductive, rigid, flat, concentric-circular thin plate, forming the topside structure of the proposed pressure sensor. The conductive, rigid, flat, concentric-circular thin plate, as a movable upper electrode plate, forms a parallel plate capacitor with the flat lower electrode plate. On application of the pressure *q*, as shown in Figure 2b, the initially flat non-conductive elastic annular membrane will deflect towards the lower electrode plate and work as an elastic deformation element in response to the applied pressure *q*, resulting in the upper electrode plate moving a distance *w_m_* (the maximum elastic deflection) from its initial position (that of the initially flat annular membrane) towards the lower electrode plate. Clearly, the movement of the upper electrode plate will result in a capacitance change in the parallel plate capacitor. Therefore, the applied pressure *q* can be determined by measuring the capacitance change caused in the parallel plate capacitor.

The topside structure of the proposed elastic-deflection-and-parallel-plate-capacitor-based pressure sensor can also be formed by a non-conductive elastic circular membrane whose central region firmly adheres to the conductive, rigid, flat, concentric-circular thin plate (such that the central region membrane will not produce elastic deformation when the upper electrode plate moves). In addition, the initial gap *g* between the upper and lower electrode plates should be far less than the diameter 2*b* of the upper and lower electrode plates, such that the fringe effect in the capacitance calculation of the parallel plate capacitor can be ignored. Therefore, the capacitance between the two parallel conductive, flat, circular thin plates with radius *b*, dielectric constant *ε*, and air gap *g-w_m_* (see Figure 2b), after neglecting the fringe effect, may be written as [25]

(1) $$C=\varepsilon \frac{\pi b^{2}}{g-w_{m}}.$$

As mentioned above, the application of a pressure *q* will result in the maximum elastic deflection *w_m_*. In other words, there is a one-to-one correspondence. Hence, *w_m_* is a continuous function of *q*, i.e., *w_m_*(*q*). Therefore, once *w_m_*(*q*) is obtained, the relationship between the pressure *q* and the capacitance *C* can be determined. We must solve for the behavior of the elastic deformation with large deflection of the annular membrane analytically, to obtain an accurate continuous function *w_m_*(*q*).

Analytical solutions for the large-deflection phenomenon of elastic membranes are available in only a few cases, due to the difficulties of analysis. However, for the design and development of elastic-deflection-based devices, accurate analytical solutions are often found to be necessary [26,27]. Our earlier work [24] failed to accurately solve the behavior of the elastic deformation with large deflection of the annular membrane (see Figure 2b) analytically. The closed-form solution presented in [24] could not meet the accuracy requirements for designing the proposed elastic-deflection-and-parallel-plate-capacitor-based pressure sensor, as it introduced too many pressure-measurement errors for the same input capacitance (i.e., under the same maximum elastic deflection *w_m_*). Therefore, the next section is devoted to the new and more refined closed-form solution for the behavior of the elastic deformation with large deflection of the annular membrane.

## 3. Refined Closed-Form Solution

**Figure 3:**
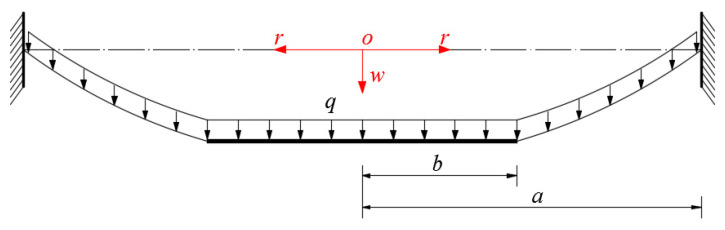
Sketch of the annular membrane under transverse loads *q*.

**Figure 4:**
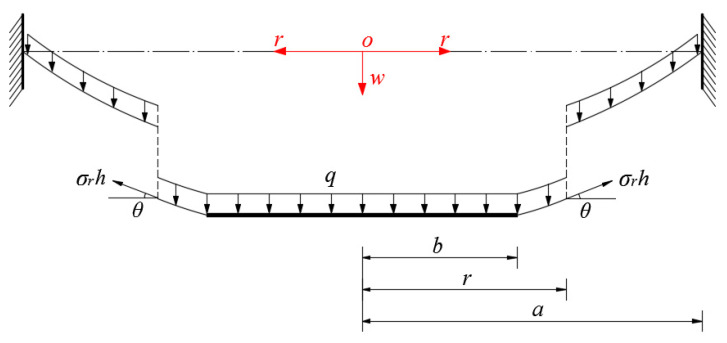
Sketch of a free body with radius *b* ≤ *r* ≤ *a*.

An initially flat, linearly elastic annular membrane with thickness *h*, outer radius *a*, inner radius *b*, Poisson’s ratio *v*, and Young’s modulus of elasticity *E* is tightly fixed at its outer edge and connected at its inner edge to a movable, weightless, rigid, concentric-circular thin plate of radius *b*, resulting in an immovable and non-deformable outer edge and a movable but non-deformable inner edge. At the same time, a uniformly distributed transverse load *q* is quasi-statically applied to the annular membrane and movable, weightless, rigid, concentric-circular thin plate, resulting in an out-of-plane displacement (deflection) of the annular membrane, as shown in Figure 3. In the figure, a cylindrical coordinate system (*r, φ, w*) is introduced, with the polar coordinate plane (*r, φ*) located in the plane in which the geometric middle plane of the initially flat annular membrane is located, and where *o* denotes the origin of the introduced cylindrical coordinate system (*r, φ, w*) (which is placed in the centroid of the geometric middle plane), *r* denotes the radial coordinate, *φ* denotes the angle coordinate (not represented in Figure 3), and *w* denotes the axial coordinate as well as the transverse displacement of the deflected membrane. A free body, a piece of annular membrane with radius *r* (*b* *≤ r* *≤ a*), is taken from the central portion of the deflected annular membrane, to study the static problem of equilibrium of this free body under the joint action of the external active force *πr*^2^*q* produced by the uniformly distributed transverse loads *q* and the reactive force 2*πrσ_r_h* produced by the membrane force *σ_r_h* acting on the boundary *r*, as shown in Figure 4, where *σ_r_* denotes the radial stress and *θ* denotes the rotation angle of the deflected annular membrane.

The so-called out-of-plane equilibrium equation can be obtained from the equilibrium condition that the resultant force in the transverse (vertical) direction is equal to zero, and is given by


(2) $$2\pi r\sigma_{r}h\sin\theta =\pi r^{2}q.$$

If the transverse displacement of the deflected annular membrane at *r* is denoted by *w*(*r*), then

(3) $$\sin\theta =1/\sqrt{1+1/\tan^{2}\theta }=1/\sqrt{1+1/{\left(-dw/dr\right)}^{2}}.$$

Substituting Equation (3) into Equation (2), the out-of-plane equilibrium equation can be written as

(4) $$2\sigma_{r}h=rq\sqrt{1+1/{\left(-dw/dr\right)}^{2}}.$$

In the horizontal direction parallel to the initially flat annular membrane, there are the actions of the radial membrane force *σ_r_h* and the circumferential membrane force *σ_t_h*, where *σ_t_* denotes the circumferential stress. Therefore, the so-called in-plane equilibrium equation may be written as

(5) $$\frac{d}{dr}(r\sigma_{r}h)-\sigma_{t}h=0.$$

If the radial strain, circumferential strain, and radial displacement are denoted by *e_r_*, *e_t_*, and *u*(*r*), respectively, then the relations between the strain and displacement, the so-called geometric equations, may be written as

(6) $$e_{r}=\frac{du}{dr}+\frac{1}{2}{\left(\frac{dw}{dr}\right)}^{2}$$

and

(7) $$e_{t}=\frac{u}{r}.$$

Moreover, the relations between the stress and strain, the so-called physical equations, are still assumed to satisfy linear elasticity

(8) $$\sigma_{r}=\frac{E}{1-\nu^{2}}(e_{r}+\nu e_{t})$$

and

(9) $$\sigma_{t}=\frac{E}{1-\nu^{2}}(e_{t}+\nu e_{r}).$$

Substituting Equations (6) and (7) into Equations (8) and (9) yields

(10) $$\sigma_{r}=\frac{E}{1-\nu^{2}}[\frac{du}{dr}+\frac{1}{2}{\left(\frac{dw}{dr}\right)}^{2}+\nu \frac{u}{r}]$$

and

(11) $$\sigma_{t}=\frac{E}{1-\nu^{2}}[\frac{u}{r}+\nu \frac{du}{dr}+\frac{\nu }{2}{\left(\frac{dw}{dr}\right)}^{2}].$$

Eliminating d*u/*d*r* from Equations (10) and (11) and further using Equation (5), it is found that

(12) $$\frac{u}{r}=\frac{1}{Eh}(\sigma_{t}h-\nu \sigma_{r}h)=\frac{1}{Eh}[\frac{d}{dr}(r\sigma_{r}h)-\nu \sigma_{r}h].$$

After substituting the *u* of Equation (12) into Equation (10), the so-called consistency equation may be written as

(13) $$r\frac{d}{dr}[\frac{1}{r}\frac{d}{dr}(r^{2}\sigma_{r})]+\frac{E}{2}{\left(\frac{dw}{dr}\right)}^{2}=0.$$

Equations (4), (5) and (13) are three equations for the solutions of *σ_r_*, *σ_t_*, and *w*. The boundary conditions for solving Equations (4), (5) and (13) are

(14) $$e_{t}=0(\mathrm{or}\;\frac{u}{r}=0)\;\mathrm{at}\;r=b,$$

(15) $$e_{t}=0(\frac{u}{r}=0)\;\mathrm{at}\;r=a$$

and

(16) w=0 at r=a.

Let us introduce the nondimensionalization

(17) $$Q=\frac{aq}{Eh},W=\frac{w}{a},S_{r}=\frac{\sigma_{r}}{E},S_{t}=\frac{\sigma_{t}}{E},\alpha =\frac{b}{a},x=\frac{r}{a},$$

and transform Equations (4), (5) and (12)–(16) into

(18) $$2S_{r}=xQ\sqrt{1+1/{\left(-dW/dx\right)}^{2}},$$

(19) $$\frac{d(xS_{r})}{dx}-S_{t}=0,$$

(20) $$\frac{u}{r}=(1-\nu )S_{r}+x\frac{dS_{r}}{dx},$$

(21) $$x^{2}\frac{d^{2}S_{r}}{dx^{2}}+3x\frac{dS_{r}}{dx}+\frac{1}{2}{\left(\frac{dW}{dx}\right)}^{2}=0,$$

(22) $$(1-\nu )S_{r}+x\frac{dS_{r}}{dx}=0\;\mathrm{at}\;x=\alpha ,$$

(23) $$(1-\nu )S_{r}+x\frac{dS_{r}}{dx}=0\;\mathrm{at}\;x=1$$

and

(24) W=0 at x=1.

From Equation (18), it is found that

(25) $${\left(-\frac{dW}{dx}\right)}^{2}=\frac{x^{2}Q^{2}}{4S_{r}^{2}-x^{2}Q^{2}}.$$

Eliminating d*W*/d*x* from Equations (21) and (25), we can obtain an equation which contains only *S_r_*:

(26) $$x^{2}\frac{d^{2}S_{r}}{dx^{2}}+3x\frac{dS_{r}}{dx}+\frac{x^{2}Q^{2}}{8S_{r}^{2}-2x^{2}Q^{2}}=0.$$

In view of the physical phenomenon that the values of stress, strain, and displacement are all finite within the range *α* ≤ *x* ≤ 1, we can expand *S_r_* and *W* into a power series of (*x*−*β*), i.e.,

(27) $$S_{r}=\sum_{i=0}^{\infty }c_{i}{\left(x-\beta \right)}^{i}$$

and

(28) $$W=\sum_{i=0}^{\infty }d_{i}{\left(x-\beta \right)}^{i},$$

where *β* = (1 + *α*)/2. For convenience we introduce *X* = *x* − *β*, then Equations (25)–(28) can be transformed into

(29) $${\left(-\frac{dW}{dx}\right)}^{2}=\frac{{\left(X+\beta \right)}^{2}Q^{2}}{4S_{r}^{2}-{\left(X+\beta \right)}^{2}Q^{2}},$$

(30) $${\left(X+\beta \right)}^{2}\frac{d^{2}S_{r}}{dX^{2}}+3(X+\beta )\frac{dS_{r}}{dX}+\frac{{\left(X+\beta \right)}^{2}Q^{2}}{8S_{r}^{2}-2{\left(X+\beta \right)}^{2}Q^{2}}=0,$$

(31) $$S_{r}=\sum_{i=0}^{\infty }c_{i}X^{i}$$

and

(32) $$W=\sum_{i=0}^{\infty }d_{i}X^{i}.$$

Substituting Equation (31) into Equation (30) and letting the sums of all coefficients of the same powers of *X* be equal to zero yields a system of equations for determining the recursion formulas for the coefficients *c_i_*. The solution to this system of equations shows that the coefficients *c_i_* (*i* = 2, 3, 4, …) can be expressed as polynomial functions with regard to the first two coefficient *c*_0_ and *c*_1_ (see Appendix A). Further, by substituting Equations (31) and (32) into Equation (29), the coefficients *d_i_* (*i* = 1, 2, 3, …) can also be expressed in terms of *c*_0_ and *c*_1_ (see Appendix B).

The remaining three coefficients *c*_0_, *c*_1_, and *d*_0_ are three undetermined constants, which depend on the specific problem addressed and can be determined by using the boundary conditions of Equations (22) and (23), as follows. Substituting Equation (31) into Equations (22) and (23) yields

(33) $$(1-\nu )\sum_{i=0}^{\infty }c_{i}{\left(\alpha -\beta \right)}^{i}+\alpha \sum_{i=1}^{\infty }ic_{i}{\left(\alpha -\beta \right)}^{i-1}=0$$

and

(34) $$(1-\nu )\sum_{i=0}^{\infty }c_{i}{\left(1-\beta \right)}^{i}+\sum_{i=1}^{\infty }ic_{i}{\left(1-\beta \right)}^{i-1}=0.$$

If all the recursion formulas for the coefficients *c_i_* in Appendix A are repeatedly substituted into Equations (33) and (34), Equations (33) and (34) will contain only *c*_0_ and *c*_1_. Therefore, the values of *c*_0_ and *c*_1_ can be determined by simultaneously solving Equations (33) and (34), and the expression for *S_r_* can be determined. Further, substituting Equation (32) into the boundary condition of Equation (24) yields

(35) $$d_{0}=-\sum_{i=1}^{\infty }d_{i}{\left(1-\beta \right)}^{i}.$$

Therefore, with the known *c*_0_ and *c*_1_, the value of *d*_0_ can finally be determined using Equation (35), and the expression for W can also be determined. The expression for *S_t_*, with the known expression of *S_r_*, can easily be determined by Equation (19).

The problem addressed here is thus solved analytically, and its closed-form solutions for stress and deflection are given. The closed-form solution for stress will be used to check whether the thin film used meets the mechanical strength required, while the closed-form solution for deflection will be used to determine the important analytical relationship between the applied pressure *q* and the maximum deflection *w_m_*.

The maximum deflection of the deflected annular membrane *w_m_* is at *x* = *α*, and from Equations (17) and (28) it may finally be written as

(36) $$w_{m}=a\sum_{i=0}^{\infty }d_{i}{\left(\frac{b-a}{2a}\right)}^{i}.$$

The maximum stress of the deflected annular membrane *σ_m_* is also at *x* = *α*, and from Equations (17) and (27) it may be written as

(37) $$\sigma_{m}=\sigma_{r}(b)=E\sum_{i=0}^{\infty }c_{i}{\left(\frac{b-a}{2a}\right)}^{i}.$$

## 4. Results and Discussion

### 4.1. Validity of the Closed-Form Solution Obtained

**Figure 5:**
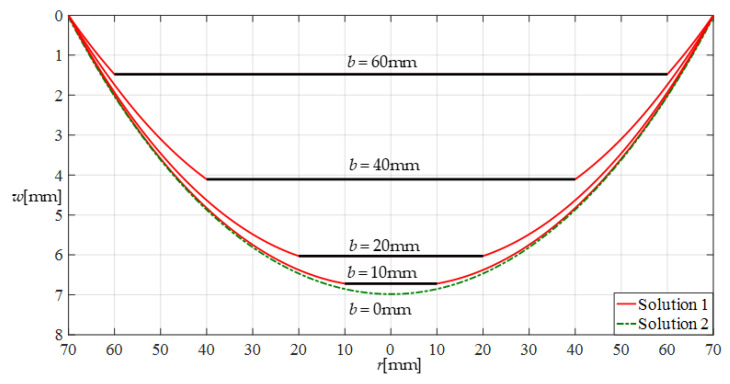
Variations of *w* with *r* when *q* = 0.0001 MPa and *b* takes different values.

Clearly, under the same transverse loads *q*, an annular membrane with outer radius *a* and inner radius *b* should have the same deflection curve as a circular membrane with radius *a* when *b*→0. Therefore, the validity of the closed-form solution given in Section 2 can be proved by examining whether the deflection curves of the annular membrane with outer radius *a* and inner radius *b* can gradually approach the deflection curve of the circular membrane with radius *a*, as the inner radius *b* gradually approaches zero. To this end, a numerical example is presented, where a circular membrane with Young’s modulus of elasticity *E* = 7.84 MPa, Poisson’s ratio *v* = 0.47, thickness *h* = 0.2 mm, and radius *a* = 70 mm, and four annular membranes with Young’s modulus of elasticity *E* = 7.84 MPa, Poisson’s ratio *v* = 0.47, thickness *h* = 0.2 mm, outer radius *a* = 70 mm, and inner radii *b* = 60 mm, 40 mm, 20 mm, and 10 mm are subjected to the same transverse loads *q* = 0.0001 MPa. The deflections of the annular membranes are calculated by using the closed-form solution obtained in Section 2, while the deflection of the circular membrane is calculated by using the closed-form solution presented in [24], which is also obtained using “sin*θ* = 1/(1 + 1/tan^2^*θ*)^1/2^” rather than “sin*θ* = tan*θ*”. The results of the deflection calculation are shown in Figure 5, where “Solution 1” refers to the closed-form solution given in Section 2, and “Solution 2” refers to the closed-form solution presented in [28]. It may be seen from Figure 5 that the deflection curves of the annular membranes gradually approach the deflection curve of the circular membrane as the inner radius *b* decreases gradually from 60 mm to 10 mm, which to some extent shows that the closed-form solution given in Section 2 is correctly derived and basically reliable.

### 4.2. Comparison of the Closed-Form Solutions before and after Improvement

**Figure 6:**
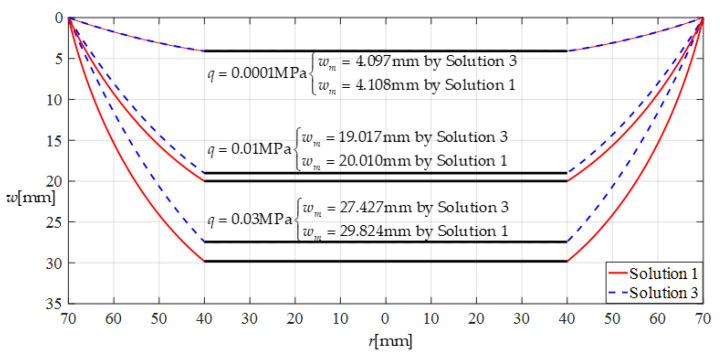
Deflection comparison under the same loads *q*.

Figure 6 shows the differences between the deflection curves calculated under the same loads *q* by the closed-form solutions before and after improvement, where the annular membrane used has Young’s modulus of elasticity *E* = 7.84 MPa, Poisson’s ratio *v* = 0.47, thickness *h* = 0.2 mm, outer radius *a* = 70 mm, and inner radius *b* = 40 mm. “Solution 1” refers to the closed-form solution after improvement (given in this paper), and “Solution 3” refers to the closed-form solution before improvement (presented in [24]). In Figure 6, the maximum deflection values for *q* = 0.0001 MPa are about 4.108 mm (calculated in Solution 1) and 4.097 mm (calculated in Solution 3), the maximum deflection values for *q* = 0.01 MPa are about 20.010 mm (Solution 1) and 19.017 mm (Solution 3), and the maximum deflection values for *q* = 0.03 MPa are about 29.824 mm (Solution 1) and 27.427 mm (Solution 3). From Figure 6 it may be seen that the solutions before and after improvement agree quite closely for the lightly loaded case and diverge slowly as the loads *q* increase. This is because the rotation angle *θ* of the annular membrane increases gradually as the loads *q* increase, and therefore, the error in the deflection calculation introduced by the closed-form solution before improvement (obtained using “sin*θ* = tan*θ*” rather than “sin*θ* = 1/(1 + 1/tan^2^*θ*)^1/2^”) increases gradually as the rotation angle *θ* increases. This means that it is necessary to use “sin*θ* = 1/(1 + 1/tan^2^*θ*)^1/2^” (rather than “sin*θ* = tan*θ*”) in the derivation of the closed-form solution.

**Figure 7:**
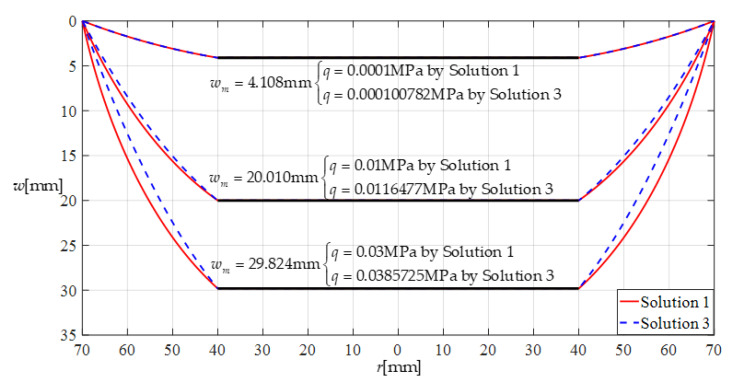
Pressure comparison under the same maximum deflection.

Clearly, if a capacitive pressure sensor based on thin film elastic deflection and a parallel plate capacitor is designed using the closed-form solution before improvement, then its pressure measurement error can be estimated directly by the error in the pressure values under the same maximum deflection *w_m_* (in order to keep the capacitance of the parallel plate capacitor the same (see Figure 1)), calculated by using the closed-form solutions before and after improvement. Figure 7 shows the pressure difference under the same maximum deflection *w_m_*, where “Solution 1” refers to the closed-form solution after improvement (presented in this paper) and “Solution 3” refers to the closed-form solution before improvement (presented in [24]). The relative error of “Solution 3” compared to “Solution 1”, that is, the pressure measurement error caused by using the closed-form solution before improvement, is about 0.782% for *w_m_* = 4.108 mm, 16.477% for *w_m_* = 20.010 mm, and 28.575% for *w_m_* = 29.824 mm. This suggests that the improvement of the closed-form solution is very important for the design of the proposed capacitive pressure sensors.

### 4.3. Numerical Calibration Based on the Closed-Form Solution Obtained

The proposed capacitive pressure sensors based on thin film elastic deflection and a parallel plate capacitor may be numerically calibrated based on the closed-form solution for the large deflection problem in Figure 2b, which was not addressed in our earlier work [24]. In this section, based on the closed-form solution given in Section 2, the numerical calibration of the proposed capacitive pressure sensors is detailed as follows.

**Figure 8:**
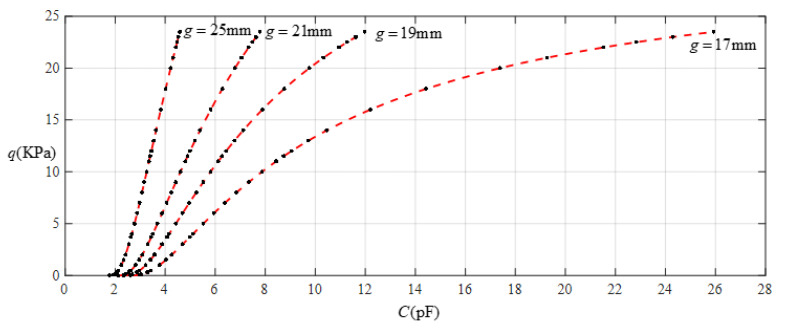
Relationship between pressure *q* and capacitance *C* when *a* = 70 mm, *b* = 40 mm, *E* = 7.84 MPa, *ν* = 0.47, and *h* = 1 mm, with values of *g* of 17 mm, 19 mm, 21 mm, and 25 mm.

Suppose that an annular elastic thin film is used as the deformable element of a pressure sensor to be calibrated, where the outer radius of the pressure sensor is *a* = 70 mm, inner radius is *b* = 40 mm, thickness is *h* = 1 mm, Young’s modulus of elasticity is *E* = 7.84 MPa, Poisson’s ratio is *v* = 0.47, and yield strength is *σ*_y_ = 2.4 MPa. The pressure-measurement range of the capacitive pressure sensor to be designed can be determined by the strength of the elastic thin film used; that is, the maximum elastic stress of the deflected annular thin film σ_m_ must be less than its yield stress *σ*_y_. Suppose that the maximum elastic stress of the deflected annular thin film is controlled at *σ*_m_ ≤ 0.7*σ*_y_ = 1.68 MPa, and the initial air gap *g* (see Figure 1) takes values of 17 mm, 19 mm, 21 mm, and 25 mm. Table 1 shows the calculation results for the maximum stress *σ*_m_, maximum deflection *w*_m_, and capacitance *C* when the pressure *q* ranges from 0 to 23.50 KPa. Figure 8 shows the relationship between pressure *q* and capacitance *C* when the initial air gap *g* takes values of 17 mm, 19 mm, 21 mm, and 25 mm.

**Figure 9:**
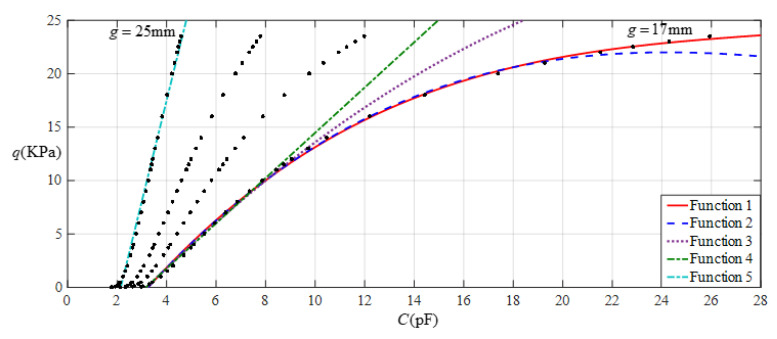
Fitting functions when *a* = 70 mm, *b* = 40 mm, *E* = 7.84 MPa, *ν* = 0.47, and *h* = 1 mm, with values of *g* of 17 mm and 25 mm.

It is often desirable that a sensor is designed with a linear input–output relationship. From Figure 8 it can be seen that the analytical relationship between output pressure *q* and input capacitance *C* can be made more linear by increasing the initial air gap *g*. This, however, will narrow the range of capacitance variation and eventually increase the output pressure per unit capacitance, in addition to increasing the edge effect in the capacitance of the parallel plate capacitor. Therefore, it is best not to do so unless it is necessary. Figure 9 shows the effect of least-squares data fitting for processing the data in Table 1, where Function 1 is a quartic function, Function 2 is a cubic function, Function 3 is a quadratic function, Function 4 is a straight line, and Function 5 is also a straight line. Functions 1−4 correspond to *g* = 17 mm, and Function 5 corresponds to *g* = 25 mm. The change in the ranges of the input capacitance *C* and the output pressure *q* and the expressions for Functions 1−5 are listed in Table 2. It can be seen from Figure 9 or Table 2 that if the ranges of input capacitance and output pressure are expected to be as large as possible and the output pressure per unit capacitance as small as possible, then the sensor must be a nonlinear sensor, that is, it must be calibrated with Function 3 or Function 2, or especially Function 1 (see Figure 9). It can be found from Table 2 that the output pressure per unit capacitance is about 2.003 KPa/pF and 8.863 KPa/pF corresponding to the two straight-line functions Function 4 and Function 5, respectively. Therefore, if an output pressure of 0.06~11 KPa can meet the design requirements, it is obvious that Function 4 should be used for linear calibration, rather than Function 5.

### 4.4. Effect of Important Parametric Variations on Input–Output Relationships

Now, let us address the effect of changes in the Young’s modulus of elasticity *E* and thickness *h* of the membranes on the input–output analytical relationship for sensors. This work has important theoretical significance for guiding the design of capacitive pressure sensors based on thin film elastic deflection and a parallel plate capacitor. By changing the Young’s modulus of elasticity *E* and thickness *h* of the membranes and carrying out a series of numerical calibrations, the variation trend of the operational pressure ranges and input–output characteristics with important parametric variations can be found. This can clarify how to appropriately prepare or select materials to achieve the desired operational pressure ranges and input–output characteristics. To this end, the annular elastic thin film used above still maintains an outer radius *a* = 70 mm, inner radius *b* = 40 mm, Poisson’s ratio *v* = 0.47, and yield strength *σ*_y_ = 2.4 MPa. In order to investigate the effect of the Young’s modulus of elasticity, the thickness of the thin film maintains *h* = 1 mm and its Young’s modulus of elasticity takes the values *E* = 5 MPa and *E* = 2.5 MPa, while in order to investigate the thickness effect, the Young’s modulus of elasticity maintains *E* = 7.84 MPa and the thickness takes the values *h* = 0.7 mm and *h* = 0.4 mm.

**Figure 10:**
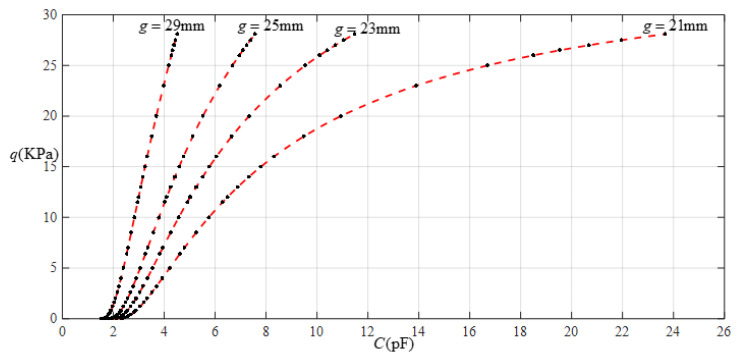
Relationship between pressure *q* and capacitance *C* when *a* = 70 mm, *b* = 40 mm, *E* = 5 MPa, *ν* = 0.47, and *h* = 1 mm, with values of *g* of 21 mm, 23 mm, 25 mm, and 29 mm.

**Figure 11:**
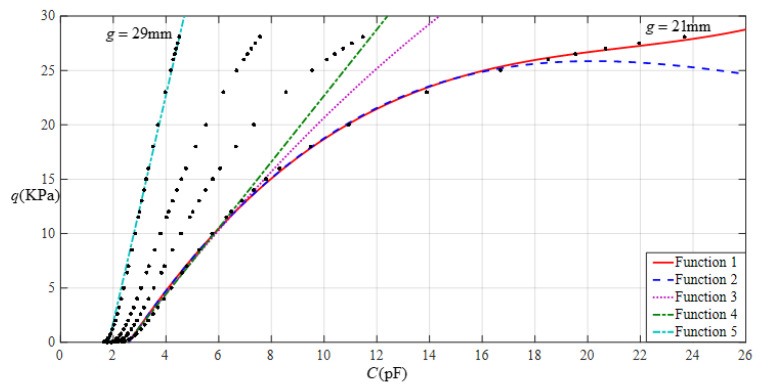
Fitting functions when *a* = 70 mm, *b* = 40 mm, *E* = 5 MPa, *ν* = 0.47, and *h* = 1 mm, with values of *g* of 21 mm and 29 mm.

Figure 10 shows the relationship between pressure *q* and capacitance *C* when *a* = 70 mm, *b* = 40 mm, *E* = 5 MPa, *ν* = 0.47, and *h* = 1 mm, with values of *g* of 21 mm, 23 mm, 25 mm, and 29 mm. Figure 11 shows the fitting functions when *a* = 70 mm, *b* = 40 mm, *E* = 5 MPa, *ν* = 0.47, and *h* = 1 mm, with values of *g* of 21 mm and 29 mm, where Functions 1−4 correspond to *g* = 21 mm, Function 5 corresponds to *g* = 29 mm, and the range of input capacitance and output pressure and the functional expressions fitted using the least squares method are listed in Table 3.

**Figure 12:**
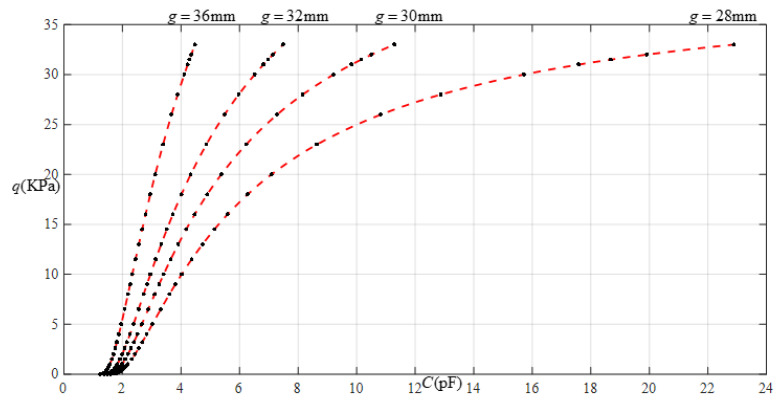
Relationship between pressure *q* and capacitance *C* when *a* = 70 mm, *b* = 40 mm, *E* = 2.5 MPa, *ν* = 0.47, and *h* = 1 mm, with values of *g* of 28 mm, 30 mm, 32 mm, and 36 mm.

**Figure 13:**
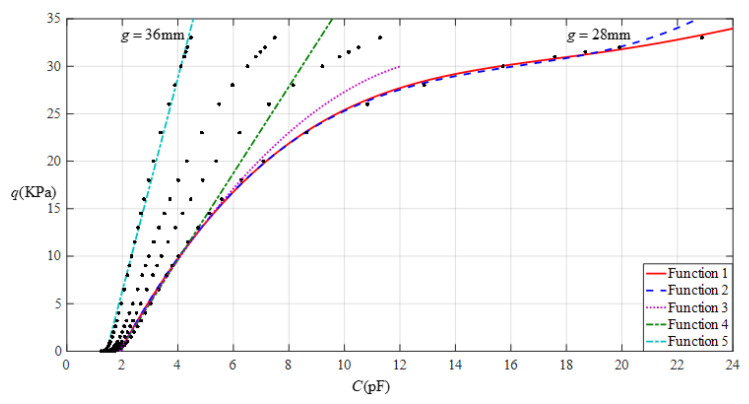
Fitting functions when *a* = 70 mm, *b* = 40 mm, *E* = 2.5 MPa, *ν* = 0.47, and *h* = 1 mm, with values of *g* of 28 mm and 36 mm.

Figure 12 shows the relationship between pressure *q* and capacitance *C* when *a* = 70 mm, *b* = 40 mm, *E* = 2.5 MPa, *ν* = 0.47, and *h* = 1 mm, with values of *g* of 28 mm, 30 mm, 32 mm, and 36 mm. Figure 13 shows the fitting functions when *a* = 70 mm, *b* = 40 mm, *E* = 2.5 MPa, *ν* = 0.47, and *h* = 1 mm, with values of *g* of 28 mm and 36 mm, where Functions 1−4 correspond to *g* = 28 mm, Function 5 corresponds to *g* = 36 mm, and the range of input capacitance and output pressure and the functional expressions fitted by the least squares method are listed in Table 4.

**Figure 14:**
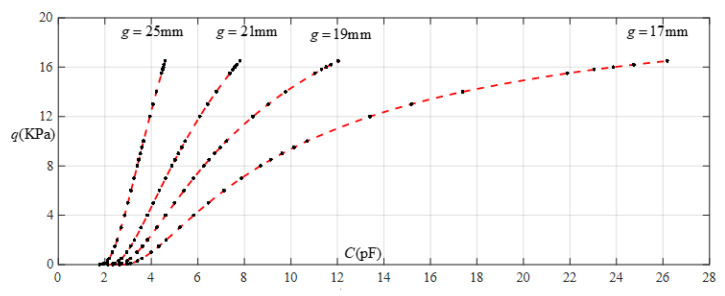
Relationship between pressure *q* and capacitance *C* when *a* = 70 mm, *b* = 40 mm, *E* = 7.84 MPa, *ν* = 0.47, and *h* = 0.7 mm, with values of *g* of 17 mm, 19 mm, 21 mm, and 25 mm.

**Figure 15:**
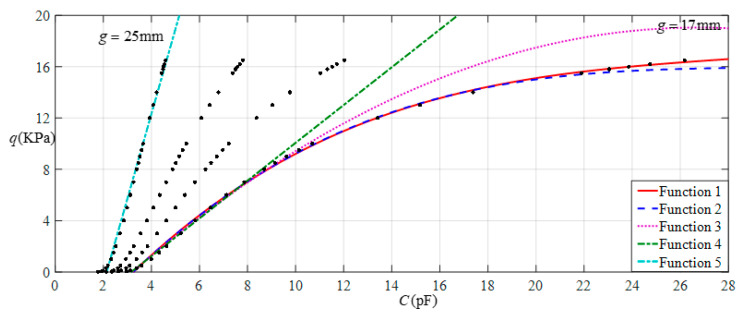
Fitting functions when *a* = 70 mm, *b* = 40 mm, *E* = 7.84 MPa, *ν* = 0.47, and *h* = 0.7 mm, with values of *g* of 17 mm and 25 mm.

Figure 14 shows the relationship between pressure *q* and capacitance *C* when *a* = 70 mm, *b* = 40 mm, *E* = 7.84 MPa, *ν* = 0.47, and *h* = 0.7 mm, with values of *g* of 17 mm, 19 mm, 21 mm, and 25 mm. Figure 15 shows the fitting functions when *a* = 70 mm, *b* = 40 mm, *E* = 7.84 MPa, *ν* = 0.47, and *h* = 0.7 mm, with values of *g* of 17 mm and 25 mm, where Functions 1−4 correspond to *g* = 17 mm, Function 5 corresponds to *g* = 25 mm, and the range of input capacitance and output pressure and the functional expressions fitted by the least squares method are listed in Table 5.

**Figure 16:**
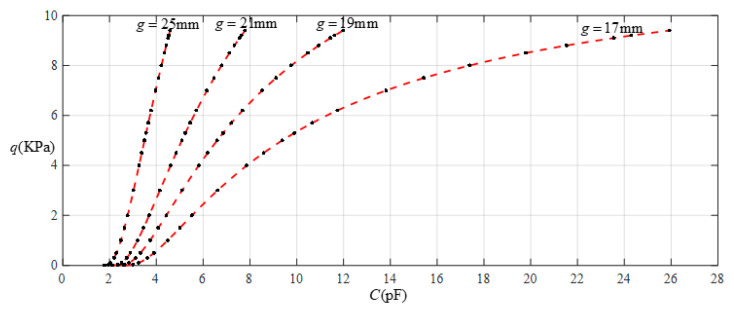
Relationship between pressure *q* and capacitance *C* when *a* = 70 mm, *b* = 40 mm, *E* = 7.84 MPa, *ν* = 0.47, and *h* = 0.4 mm, with values of *g* of 17 mm, 19 mm, 21 mm, and 25 mm.

**Figure 17:**
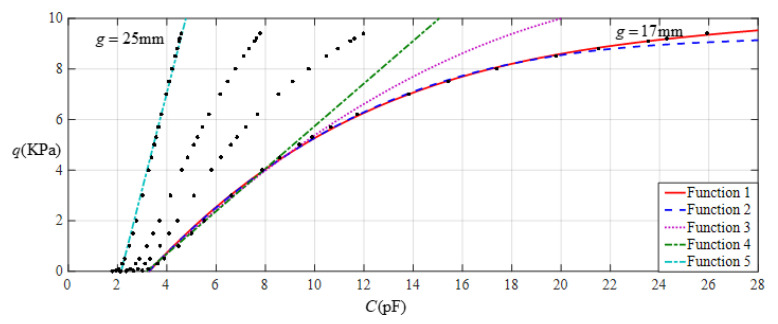
Fitting functions when *a* = 70 mm, *b* = 40 mm, *E* = 7.84 MPa, *ν* = 0.47, and *h* = 0.4 mm, with values of *g* of 17 mm and 25 mm.

Figure 16 shows the relationship between pressure *q* and capacitance *C* when *a* = 70 mm, *b* = 40 mm, *E* = 7.84 MPa, *ν* = 0.47, and *h* = 0.4 mm, with values of *g* of 17 mm, 19 mm, 21 mm, and 25 mm. Figure 17 shows the fitting functions when *a* = 70 mm, *b* = 40 mm, *E* = 7.84 MPa, *ν* = 0.47, and *h* = 0.4 mm, with values of *g* of 17 mm and 25 mm, where Functions 1−4 correspond to *g* = 17 mm, Function 5 corresponds to *g* = 25 mm, and the range of input capacitance and output pressure and the functional expressions fitted by the least squares method are listed in Table 6.

Now, the effect of varying the Young’s modulus of elasticity *E* and thickness *h* of membrane on the input–output relationships can be summarized as follows.

**Figure 18:**
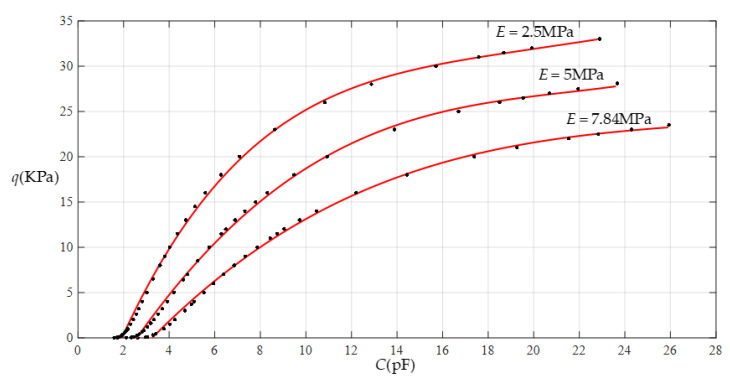
Effect of varying elastic modulus *E* on Function 1 (fitting with a quartic function).

**Figure 19:**
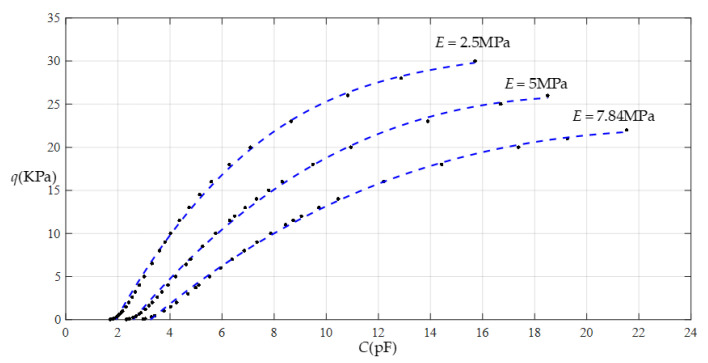
Effect of varying elastic modulus *E* on Function 2 (fitting with a cubic function).

**Figure 20:**
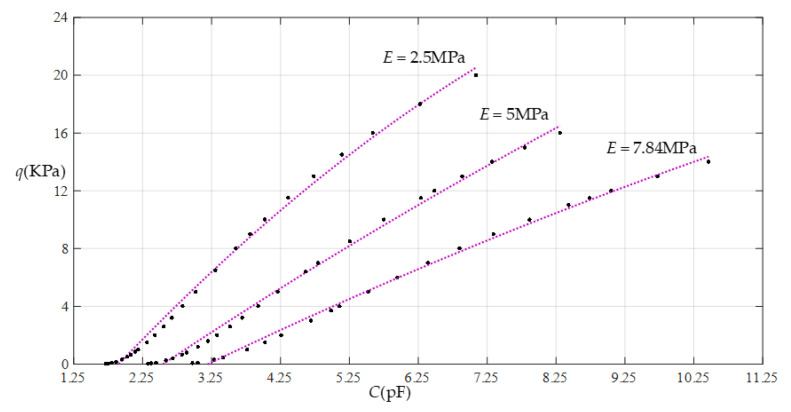
Effect of varying elastic modulus *E* on Function 3 (fitting with a quadratic function).

**Figure 21:**
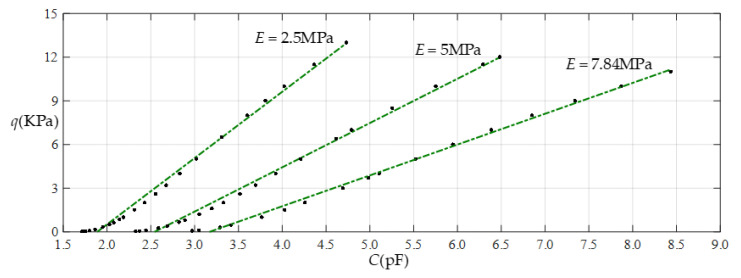
Effect of varying elastic modulus *E* on Function 4 (fitting with a straight line).

When the thickness *h* of the membrane remains constant and the Young’s modulus of elasticity *E* is changed, it can be seen from Table 2, Table 3 and Table 4 that, with a decrease in the Young’s modulus of elasticity *E*, the range of the output pressure increases, while the range of the input capacitance decreases. This means that large operational pressure ranges require the use of thin films with a low Young’s modulus of elasticity *E*, while large operational capacitance ranges require the use of thin films with a high Young’s modulus of elasticity *E*. In addition, the increase in the output pressure range is a maximum in the case of Function 1 (that is, in the case of nonlinear fitting with a quartic function) and the reduction in the input capacitance range is a maximum in the case of Function 4 (that is, in the case of linear fitting), which can be seen more clearly in Figure 18, Figure 19, Figure 20 and Figure 21.

**Figure 22:**
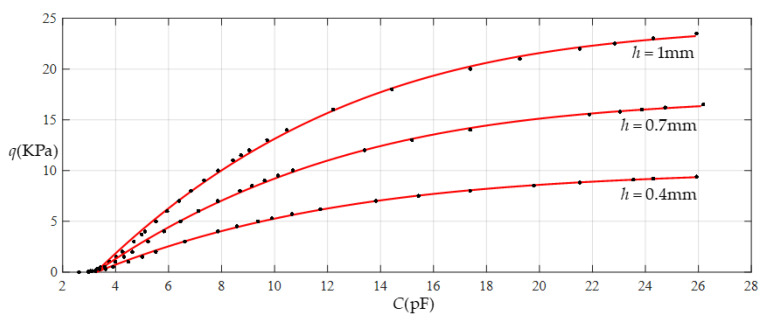
Effect of varying thickness *h* on Function 1 (fitting with a quartic function).

**Figure 23:**
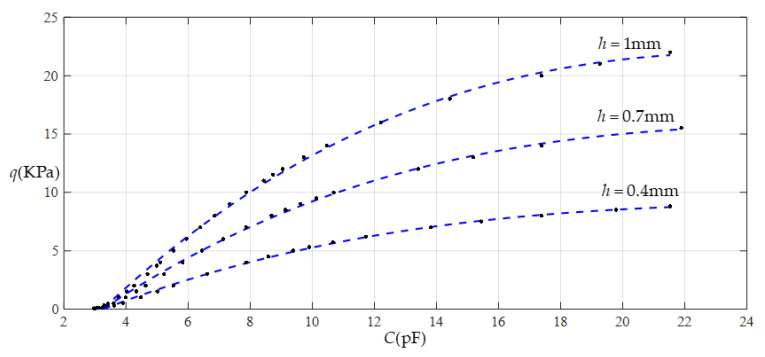
Effect of varying thickness *h* on Function 2 (fitting with a cubic function).

**Figure 24:**
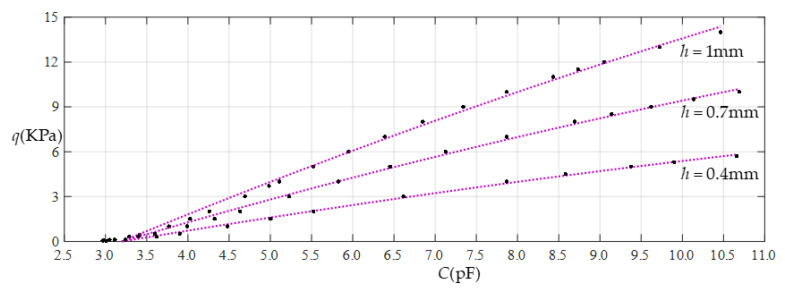
Effect of varying thickness *h* on Function 3 (fitting with a quadratic function).

**Figure 25:**
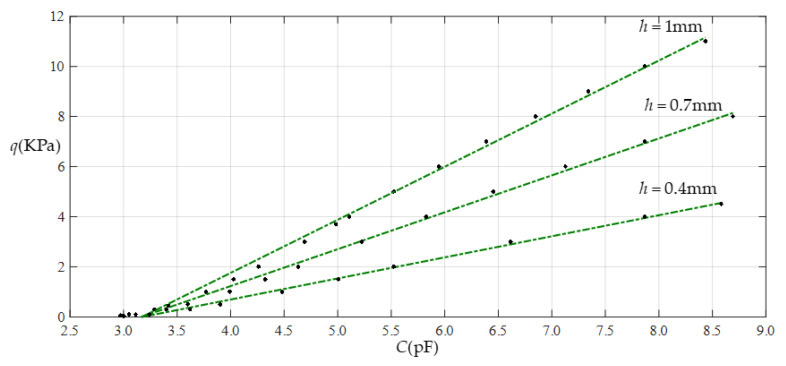
Effect of varying thickness *h* on Function 4 (fitting with a straight line).

When Young’s modulus of elasticity *E* remains constant and the thickness *h* of the membrane is changed, it can be seen from Table 2, Table 5 and Table 6 that, with the decrease in the thickness *h*, the range of the output pressure decreases, while the range of the input capacitance basically remains constant. This means that large operational pressure ranges require the use of thin films with a large thickness, while operational capacitance ranges are largely unaffected by the thickness of the thin films. In addition, the reduction in the output pressure range is a maximum in the case of Function 1 (that is, in the case of nonlinear fitting with a quartic function), which can be seen more clearly in Figure 22, Figure 23, Figure 24 and Figure 25.

## 5. Concluding Remarks

In this study, the capacitive pressure sensor based on thin film elastic deflection and a parallel plate capacitor, which was proposed in our earlier work [24], was revisited and theoretically improved. The following conclusions can be drawn from this study.

The deflection curves of the annular membrane with outer radius *a* and inner radius *b*, which are calculated using the closed-form solution given in Section 2, can gradually approach the deflection curve of the circular membrane with radius *a*, as the inner radius *b* gradually approaches zero, showing that the closed-form solution given in Section 2 is correctly derived and basically reliable.

The numerical comparison of the closed-form solution given in this paper with the one given in [24] shows that the closed-form solution given in this paper is computationally more accurate than the previous one given in [24], especially when the rotation angle *θ* of the annular membrane is relatively large or as the pressure *q* increases. This provides a reliable theory for designing the proposed capacitive pressure sensors based on thin film elastic deflection and a parallel plate capacitor.

When designing capacitive pressure sensors based on thin film elastic deflection and a parallel plate capacitor, the desired relationship between input capacitance and output pressure can be satisfied by changing the thickness *h* of the selected thin film or by selecting another thin film with a different Young’s modulus of elasticity *E*. A decrease in the Young’s modulus of elasticity *E* can increase the range of the output pressure and decrease the range of the input capacitance, while a decrease in the thickness *h* can decrease the range of the output pressure but has little effect on the range of the input capacitance.

## Figures and Tables

**Table 1:** The calculation results when *a* = 70 mm, *b* = 40 mm, *E* = 7.84 MPa, *ν* = 0.47, and *h* = 1 mm, with values of *g* of 17 mm, 19 mm, 21 mm, and 25 mm.

*q*/KPa	*σ*_m_/MPa	*w*_m_/mm	*C*/pF			
			*g* = 17 mm	*g* = 19 mm	*g* = 21 mm	*g* = 25 mm
0.00	0.00	0.000	2.619	2.344	2.121	1.781
0.06	0.03	2.022	2.973	2.623	2.346	1.938
0.10	0.04	2.398	3.050	2.682	2.394	1.970
0.30	0.08	3.462	3.289	2.866	2.539	2.068
0.45	0.11	3.965	3.416	2.962	2.614	2.117
1.00	0.19	5.184	3.769	3.223	2.816	2.247
1.50	0.25	5.942	4.027	3.410	2.957	2.337
2.00	0.30	6.548	4.260	3.576	3.081	2.413
3.00	0.40	7.511	4.693	3.876	3.301	2.546
3.70	0.46	8.066	4.984	4.073	3.443	2.630
4.00	0.49	8.283	5.108	4.155	3.502	2.664
5.00	0.57	8.938	5.523	4.425	3.692	2.772
6.00	0.64	9.512	5.947	4.694	3.876	2.875
7.00	0.72	10.029	6.388	4.964	4.059	2.974
8.00	0.79	10.500	6.851	5.239	4.241	3.071
9.00	0.85	10.935	7.342	5.521	4.424	3.166
10.00	0.92	11.340	7.867	5.813	4.610	3.260
11.00	0.98	11.720	8.434	6.117	4.799	3.353
11.50	1.01	11.902	8.735	6.274	4.895	3.400
12.00	1.05	12.079	9.049	6.434	4.992	3.446
13.00	1.11	12.420	9.723	6.767	5.190	3.540
14.00	1.17	12.745	10.464	7.118	5.394	3.633
16.00	1.28	13.352	12.208	7.885	5.823	3.823
18.00	1.40	13.914	14.431	8.756	6.285	4.017
20.00	1.51	14.439	17.385	9.762	6.787	4.216
21.00	1.56	14.689	19.266	10.329	7.056	4.319
22.00	1.62	14.931	21.526	10.945	7.338	4.423
22.50	1.64	15.050	22.837	11.274	7.484	4.475
23.00	1.67	15.167	24.297	11.618	7.635	4.529
23.50	1.68	15.283	25.933	11.980	7.789	4.583

**Table 2:** The ranges of pressure *q* and capacitance *C* and the expressions for the fitting functions in Figure 9.

Functions	Pressure *q*/KPa	Capacitance *C*/pF	Functional Expressions
Function 1	0.06~23.5	2.973~25.933	*q* = −9.4543 + 3.2678*C* − 0.1174*C^2^* + 1.6969 × 10^−3^*C*^3^ − 5.9 860 × 10^−6^*C*^4^
Function 2	0.06~22	2.973~21.526	*q* = −9.1345 + 3.1225*C* − 9.8607 × 10^−2^*C*^2^ + 9.4000 × 10^−4^*C*^3^
Function 3	0.06~14	2.973~10.464	*q* = −7.7200 + 2.5500*C* − 4.1960 × 10^−2^*C*^2^
Function 4	0.06~11	2.973~8.434	*q* = −6.7190 + 2.1193*C*
Function 5	0.06~23.5	1.938~4.583	*q* = −20.2787 + 9.4467*C*

Note: Average sums of fitting error squares for Functions 1−5 are 0.0927, 0.0949, 0.0694, 0.0473, and 0.5593, respectively.

**Table 3:** The ranges of pressure *q* and capacitance *C* and the expressions for the fitting functions in Figure 11.

Functions	Pressure *q*/KPa	Capacitance *C*/pF	Functional Expressions
Function 1	0.03~28.1	2.327~23.677	*q* = −10.387 + 4.4539*C* − 1.7499 × 10^−1^ *C^2^* + 1.8465 × 10^−3^*C*^3^ + 2.0127 × 10^−5^*C*^4^
Function 2	0.03~26	2.327~18.503	*q* = −10.320 + 4.4173*C* − 0.1713*C*^2^ + 2.0446 × 10^−3^*C*^3^
Function 3	0.03~16	2.327~8.308	*q* = −8.4201 + 3.4455*C* − 5.3760 × 10^−2^*C*^2^
Function 4	0.03~12	2.327~6.484	*q* = −7.7372 + 3.0429*C*
Function 5	0.03~28.1	1.641~4.507	*q* = −19.0409 + 10.4528*C*

Note: Average sums of fitting error squares for Functions 1−5 are 0.1565, 0.1714, 0.1218, 0.0924, and 0.5301, respectively.

**Table 4:** The ranges of pressure *q* and capacitance *C* and the expressions for the fitting functions in Figure 13.

Function	Pressure *q*/KPa	Capacitance *C*/pF	Function Expression
Function 1	0.02~33	1.716~22.884	*q* = −12.084 + 7.1260*C* − 0.4660*C*^2^ + 1.4223 × 10^−2^*C*^3^−1.6188 × 10^−4^*C*^4^
Function 2	0.02~30	1.716~15.710	*q* = −11.667 + 6.7954*C* − 0.3895*C*^2^ − 7.9564 × 10^−3^*C*^3^
Function 3	0.02~20	1.716~7.089	*q* = −10.232 + 5.7650*C* − 0.2012*C*^2^
Function 4	0.02~13	1.716~4.734	*q* = −8.6055 + 4.5583*C*
Function 5	0.02~33	1.312~4.477	*q* = −16.2264 + 11.2672*C*

Note: Average sums of fitting error squares for Functions 1−5 are 0.2123, 0.2276, 0.1922, 0.1251, and 0.5426, respectively.

**Table 5:** The ranges of pressure *q* and capacitance *C* and the expressions for the fitting functions in Figure 15.

Functions	Pressure *q*/KPa	Capacitance *C*/pF	Functional Expressions
Function 1	0.04~16.5	2.967~26.183	*q* = −6.6373 + 2.2999*C* − 8.3342 × 10^−2^*C*^2^ + 1.2233 × 10^−3^*C*^3^ − 4.3521 × 10^−6^*C*^4^
Function 2	0.04~15.5	2.967~21.887	*q* = −6.5162 + 2.2415*C* − 7.5228 × 10^−2^*C*^2^ + 8.4881 × 10^−4^*C*^3^
Function 3	0.04~10	2.967~10.690	*q* = −5.5317 + 1.8389*C* − 3.4383 × 10^−2^*C*^2^
Function 4	0.04~8	2.967~8.691	*q* = −4.6602 + 1.4732*C*
Function 5	0.04~16.5	1.935~4.590	*q* = −14.1664 + 6.6212*C*

Note: Average sums of fitting error squares for Functions 1−5 are 0.0413, 0.0468, 0.0351, 0.0260, and 0.2448, respectively.

**Table 6:** The ranges of pressure *q* and capacitance *C* and the expressions for the fitting functions in Figure 17.

Functions	Pressure *q*/KPa	Capacitance *C*/pF	Functional Expressions
Function 1	0.03~9.4	3.004~25.933	*q* = −3.9805 + 1.3871*C*−5.6008 × 10^−2^*C*^2^ + 1.0509 × 10^−3^*C*^3^−7.3096 × 10^−6^*C*^4^
Function 2	0.03~8.8	3.004~21.526	*q* = −3.7904 + 1.3020*C*−4.4844 × 10^−2^*C*^2^ + 5.2974 × 10^−4^*C*^3^
Function 3	0.03~5.7	3.004~10.661	*q* = −3.1774 + 1.0531*C*−1.9734 × 10^−2^*C*^2^
Function 4	0.03~4.5	3.004~8.583	*q* = −2.6689 + 0.8414*C*
Function 5	0.03~9.4	1.951~4.583	*q* = −8.1551 + 3.8048*C*

Note: Average sums of fitting error squares for Functions 1−5 are 0.0120, 0.0127, 0.0107, 0.0071, and 0.0669, respectively.

## Data Availability

Not applicable.

## Appendix A

$$c_{2}=-\frac{6Q^{2}\beta^{2}c_{1}-Q^{2}\beta -24c_{0}{}^{2}c_{1}}{4\beta (Q^{2}\beta^{2}-4c_{0}{}^{2})},$$

$$c_{3}=-\frac{14Q^{2}\beta^{3}c_{2}+9Q^{2}\beta^{2}c_{1}-16\beta^{2}c_{0}c_{1}c_{2}-40\beta c_{0}{}^{2}c_{2}-24\beta c_{0}c_{1}{}^{2}-Q^{2}\beta -12c_{0}{}^{2}c_{1}}{6\beta^{2}(Q^{2}\beta^{2}-4c_{0}{}^{2})},$$

$$\begin{array}{l} c_{4}=-\frac{1}{24\beta^{2}(Q^{2}\beta^{2}-4c_{0}{}^{2})}(66Q^{2}\beta^{3}c_{3}+60Q^{2}\beta^{2}c_{2}-96\beta^{2}c_{0}c_{1}c_{3}-32\beta^{2}c_{0}c_{2}{}^{2}\\ -16\beta^{2}c_{1}{}^{2}c_{2}+18Q^{2}\beta c_{1}-168\beta c_{0}{}^{2}c_{3}-208\beta c_{0}c_{1}c_{2}-24\beta c_{1}{}^{3}-64c_{0}{}^{2}c_{2}\\ -48c_{0}c_{1}{}^{2}-Q^{2}) \end{array},$$

$$\begin{array}{l} c_{5}=-\frac{1}{20\beta^{2}(Q^{2}\beta^{2}-4c_{0}{}^{2})}(60Q^{2}\beta^{3}c_{4}+63Q^{2}\beta^{2}c_{3}-96\beta^{2}c_{0}c_{1}c_{4}-64\beta^{2}c_{0}c_{2}c_{3}\\ -24\beta^{2}c_{1}{}^{2}c_{3}-16\beta^{2}c_{1}c_{2}{}^{2}+26Q^{2}\beta c_{2}-144\beta c_{0}{}^{2}c_{4}-192\beta c_{0}c_{1}c_{3}-80\beta c_{0}c_{2}{}^{2}\\ -64\beta c_{1}{}^{2}c_{2}+3Q^{2}c_{1}-60c_{0}{}^{2}c_{3}-88c_{0}c_{1}c_{2}-12c_{1}{}^{3}) \end{array},$$

$$\begin{array}{l} c_{6}=-\frac{1}{30\beta^{2}(Q^{2}\beta^{2}-4c_{0}{}^{2})}(95Q^{2}\beta^{3}c_{5}+108Q^{2}\beta^{2}c_{4}-160\beta^{2}c_{0}c_{1}c_{5}-112\beta^{2}c_{0}c_{2}c_{4}\\ -48\beta^{2}c_{0}c_{3}{}^{2}-48\beta^{2}c_{1}{}^{2}c_{4}-64\beta^{2}c_{1}c_{2}c_{3}-8\beta^{2}c_{2}{}^{3}+51Q^{2}\beta c_{3}-220\beta c_{0}{}^{2}c_{5}\\ -312\beta c_{0}c_{1}c_{4}-248\beta c_{0}c_{2}c_{3}-108\beta c_{1}{}^{2}c_{3}-92\beta c_{1}c_{2}{}^{2}+8Q^{2}c_{2}-96c_{0}{}^{2}c_{4}-144c_{0}c_{1}c_{3}\\ -64c_{0}c_{2}{}^{2}-56c_{1}{}^{2}c_{2}) \end{array},$$

$$\begin{array}{l} c_{7}=-\frac{1}{42\beta^{2}(Q^{2}\beta^{2}-4c_{0}{}^{2})}(138Q^{2}\beta^{3}c_{6}+165Q^{2}\beta^{2}c_{5}-240\beta^{2}c_{0}c_{1}c_{6}-176\beta^{2}c_{0}c_{2}c_{5}\\ -144\beta^{2}c_{0}c_{3}c_{4}-80\beta^{2}c_{1}{}^{2}c_{5}-112\beta^{2}c_{1}c_{2}c_{4}-48\beta^{2}c_{1}c_{3}{}^{2}-40\beta^{2}c_{2}{}^{2}c_{3}+84Q^{2}\beta c_{4}\\ -312\beta c_{0}{}^{2}c_{6}-464\beta c_{0}c_{1}c_{5}-368\beta c_{0}c_{2}c_{4}-168\beta c_{0}c_{3}{}^{2}-168\beta c_{1}{}^{2}c_{4}-272\beta c_{1}c_{2}c_{3}\\ -40\beta c_{2}{}^{3}+15Q^{2}c_{3}-140c_{0}{}^{2}c_{5}-216c_{0}c_{1}c_{4}-184c_{0}c_{2}c_{3}-84c_{1}{}^{2}c_{3}-76c_{1}c_{2}{}^{2}) \end{array},$$

$$\begin{array}{cc} \; & c_{8}=-\frac{1}{56\beta^{2}\left(Q^{2}\beta^{2}-4c_{0}{}^{2}\right)}\left(189Q^{2}\beta^{3}c_{7}+234Q^{2}\beta^{2}c_{6}-336\beta^{2}c_{0}c_{1}c_{7}-256\beta^{2}c_{0}c_{2}c_{6}\right.\\ \; & -208\beta^{2}c_{0}c_{3}c_{5}-96\beta^{2}c_{0}c_{4}{}^{2}-120\beta^{2}c_{1}{}^{2}c_{6}-176\beta^{2}c_{1}c_{2}c_{5}-144\beta^{2}c_{1}c_{3}c_{4}-64\beta^{2}c_{2}{}^{2}c_{4}\\ \; & -56\beta^{2}c_{2}c_{3}{}^{2}+125Q^{2}\beta c_{5}-420\beta c_{0}{}^{2}c_{7}-648\beta c_{0}c_{1}c_{6}-520\beta c_{0}c_{2}c_{5}-456\beta c_{0}c_{3}c_{4}\\ \; & -244\beta c_{1}{}^{2}c_{5}-392\beta c_{1}c_{2}c_{4}-180\beta c_{1}c_{3}{}^{2}-164\beta c_{2}{}^{2}c_{3}+24Q^{2}c_{4}-192c_{0}{}^{2}c_{6}\\ \; & \left.-304c_{0}c_{1}c_{5}-256c_{0}c_{2}c_{4}-120c_{0}c_{3}{}^{2}-120c_{1}{}^{2}c_{4}-208c_{1}c_{2}c_{3}-32c_{2}{}^{3}\right) \end{array},$$

$$\begin{array}{cc} \; & c_{9}=-\frac{1}{72\beta^{2}\left(Q^{2}\beta^{2}-4c_{0}{}^{2}\right)}\left(248Q^{2}\beta^{3}c_{8}+315Q^{2}\beta^{2}c_{7}-448\beta^{2}c_{0}c_{1}c_{8}\right.\\ \; & -352\beta^{2}c_{0}c_{2}c_{7}-288\beta^{2}c_{0}c_{3}c_{6}-256\beta^{2}c_{0}c_{4}c_{5}-168\beta^{2}c_{1}{}^{2}c_{7}-256\beta^{2}c_{1}c_{2}c_{6}\\ \; & -208\beta^{2}c_{1}c_{3}c_{5}-96\beta^{2}c_{1}c_{4}{}^{2}-96\beta^{2}c_{2}{}^{2}c_{5}-160\beta^{2}c_{2}c_{3}c_{4}-24\beta^{2}c_{3}{}^{3}\\ \; & +174Q^{2}\beta c_{6}-544\beta c_{0}{}^{2}c_{8}-864\beta c_{0}c_{1}c_{7}-704\beta c_{0}c_{2}c_{6}-608\beta c_{0}c_{3}c_{5}\\ \; & -288\beta c_{0}c_{4}{}^{2}-336\beta c_{1}{}^{2}c_{6}-544\beta c_{1}c_{2}c_{5}-480\beta c_{1}c_{3}c_{4}-224\beta c_{2}{}^{2}c_{4}\\ \; & -208\beta c_{2}c_{3}{}^{2}+35Q^{2}c_{5}-252c_{0}{}^{2}c_{7}-408c_{0}c_{1}c_{6}-344c_{0}c_{2}c_{5}-312c_{0}c_{3}c_{4}\\ \; & \left.-164c_{1}{}^{2}c_{5}-280c_{1}c_{2}c_{4}-132c_{1}c_{3}{}^{2}-124c_{2}{}^{2}c_{3}\right) \end{array},$$

$$\begin{array}{cc} \; & c_{10}=-\frac{1}{90\beta^{2}\left(Q^{2}\beta^{2}-4c_{0}{}^{2}\right)}\left(315Q^{2}\beta^{3}c_{9}+408Q^{2}\beta^{2}c_{8}-576\beta^{2}c_{0}c_{1}c_{9}\right.\\ \; & -464\beta^{2}c_{0}c_{2}c_{8}-384\beta^{2}c_{0}c_{3}c_{7}-336\beta^{2}c_{0}c_{4}c_{6}-160\beta^{2}c_{0}c_{5}{}^{2}-224\beta^{2}c_{1}{}^{2}c_{8}\\ \; & -352\beta^{2}c_{1}c_{2}c_{7}-288\beta^{2}c_{1}c_{3}c_{6}-256\beta^{2}c_{1}c_{4}c_{5}-136\beta^{2}c_{2}{}^{2}c_{6}-224\beta^{2}c_{2}c_{3}c_{5}\\ \; & -104\beta^{2}c_{2}c_{4}{}^{2}-96\beta^{2}c_{3}{}^{2}c_{4}+231Q^{2}\beta c_{7}-684\beta c_{0}{}^{2}c_{9}-1112\beta c_{0}c_{1}c_{8}\\ \; & -920\beta c_{0}c_{2}c_{7}-792\beta c_{0}c_{3}c_{6}-728\beta c_{0}c_{4}c_{5}-444\beta c_{1}{}^{2}c_{7}-728\beta c_{1}c_{2}c_{6}\\ \; & -632\beta c_{1}c_{3}c_{5}-300\beta c_{1}c_{4}{}^{2}-300\beta c_{2}{}^{2}c_{5}-536\beta c_{2}c_{3}c_{4}-84\beta c_{3}{}^{3}+48Q^{2}c_{6}\\ \; & -320c_{0}{}^{2}c_{8}-528c_{0}c_{1}c_{7}-448c_{0}c_{2}c_{6}-400c_{0}c_{3}c_{5}-192c_{0}c_{4}{}^{2}-216c_{1}{}^{2}c_{6}\\ \; & \left.-368c_{1}c_{2}c_{5}-336c_{1}c_{3}c_{4}-160c_{2}{}^{2}c_{4}-152c_{2}c_{3}{}^{2}\right) \end{array},$$

$$\begin{array}{cc} \; & c_{11}=-\frac{1}{110\beta^{2}\left(Q^{2}\beta^{2}-4c_{0}{}^{2}\right)}\left(390Q^{2}\beta^{3}c_{10}+513Q^{2}\beta^{2}c_{9}-720\beta^{2}c_{0}c_{1}c_{10}\right.\\ \; & -592\beta^{2}c_{0}c_{2}c_{9}-496\beta^{2}c_{0}c_{3}c_{8}-432\beta^{2}c_{0}c_{4}c_{7}-400\beta^{2}c_{0}c_{5}c_{6}-288\beta^{2}c_{1}{}^{2}c_{9}\\ \; & -464\beta^{2}c_{1}c_{2}c_{8}-384\beta^{2}c_{1}c_{3}c_{7}-336\beta^{2}c_{1}c_{4}c_{6}-160\beta^{2}c_{1}c_{5}{}^{2}-184\beta^{2}c_{2}{}^{2}c_{7}\\ \; & -304\beta^{2}c_{2}c_{3}c_{6}-272\beta^{2}c_{2}c_{4}c_{5}-128\beta^{2}c_{3}{}^{2}c_{5}-120\beta^{2}c_{3}c_{4}{}^{2}+296Q^{2}\beta c_{8}\\ \; & -840\beta c_{0}{}^{2}c_{10}-1392\beta c_{0}c_{1}c_{9}-1168\beta c_{0}c_{2}c_{8}-1008\beta c_{0}c_{3}c_{7}-912\beta c_{0}c_{4}c_{6}\\ \; & -440\beta c_{0}c_{5}{}^{2}-568\beta c_{1}{}^{2}c_{8}-944\beta c_{1}c_{2}c_{7}-816\beta c_{1}c_{3}c_{6}-752\beta c_{1}c_{4}c_{5}\\ \; & -392\beta c_{2}{}^{2}c_{6}-688\beta c_{2}c_{3}c_{5}-328\beta c_{2}c_{4}{}^{2}-312\beta c_{3}{}^{2}c_{4}+63Q^{2}c_{7}-396c_{0}{}^{2}c_{9}\\ \; & -664c_{0}c_{1}c_{8}-568c_{0}c_{2}c_{7}-504c_{0}c_{3}c_{6}-472c_{0}c_{4}c_{5}-276c_{1}{}^{2}c_{7}-472c_{1}c_{2}c_{6}\\ \; & \left.-424c_{1}c_{3}c_{5}-204c_{1}c_{4}{}^{2}-204c_{2}{}^{2}c_{5}-376c_{2}c_{3}c_{4}-60c_{3}{}^{3}\right) \end{array},$$

$$\begin{array}{cc} \; & c_{12}=-\frac{1}{132\beta^{2}\left(Q^{2}\beta^{2}-4c_{0}{}^{2}\right)}\left(473Q^{2}\beta^{3}c_{11}+630Q^{2}\beta^{2}c_{10}-880\beta^{2}c_{0}c_{1}c_{11}\right.\\ \; & -736\beta^{2}c_{0}c_{2}c_{10}-624\beta^{2}c_{0}c_{3}c_{9}-544\beta^{2}c_{0}c_{4}c_{8}-496\beta^{2}c_{0}c_{5}c_{7}-240\beta^{2}c_{0}c_{6}{}^{2}\\ \; & -360\beta^{2}c_{1}{}^{2}c_{10}-592\beta^{2}c_{1}c_{2}c_{9}-496\beta^{2}c_{1}c_{3}c_{8}-432\beta^{2}c_{1}c_{4}c_{7}-400\beta^{2}c_{1}c_{5}c_{6}\\ \; & -240\beta^{2}c_{2}{}^{2}c_{8}-400\beta^{2}c_{2}c_{3}c_{7}-352\beta^{2}c_{2}c_{4}c_{6}-168\beta^{2}c_{2}c_{5}{}^{2}-168\beta^{2}c_{3}{}^{2}c_{6}\\ \; & -304\beta^{2}c_{3}c_{4}c_{5}-48\beta^{2}c_{4}{}^{3}+369Q^{2}\beta c_{9}-1012\beta c_{0}{}^{2}c_{11}-1704\beta c_{0}c_{1}c_{10}\\ \; & -1448\beta c_{0}c_{2}c_{9}-1256\beta c_{0}c_{3}c_{8}-1128\beta c_{0}c_{4}c_{7}-1064\beta c_{0}c_{5}c_{6}-708\beta c_{1}{}^{2}c_{9}\\ \; & -1192\beta c_{1}c_{2}c_{8}-1032\beta c_{1}c_{3}c_{7}-936\beta c_{1}c_{4}c_{6}-452\beta c_{1}c_{5}{}^{2}-500\beta c_{2}{}^{2}c_{7}\\ \; & -872\beta c_{2}c_{3}c_{6}-808\beta c_{2}c_{4}c_{5}-388\beta c_{3}{}^{2}c_{5}-372\beta c_{3}c_{4}{}^{2}+80Q^{2}c_{8}\\ \; & -480c_{0}{}^{2}c_{10}-816c_{0}c_{1}c_{9}-704c_{0}c_{2}c_{8}-624c_{0}c_{3}c_{7}-576c_{0}c_{4}c_{6}-280c_{0}c_{5}{}^{2}\\ \; & -344c_{1}{}^{2}c_{8}-592c_{1}c_{2}c_{7}-528c_{1}c_{3}c_{6}-496c_{1}c_{4}c_{5}-256c_{2}{}^{2}c_{6}-464c_{2}c_{3}c_{5}\\ \; & \left.-224c_{2}c_{4}{}^{2}-216c_{3}{}^{2}c_{4}\right) \end{array},$$

$$\begin{array}{cc} \; & c_{13}=-\frac{1}{156\beta^{2}\left(Q^{2}\beta^{2}-4c_{0}{}^{2}\right)}\left(564Q^{2}\beta^{3}c_{12}+759Q^{2}\beta^{2}c_{11}-1056\beta^{2}c_{0}c_{1}c_{12}\right.\\ \; & -896\beta^{2}c_{0}c_{2}c_{11}-768\beta^{2}c_{0}c_{3}c_{10}-672\beta^{2}c_{0}c_{4}c_{9}-608\beta^{2}c_{0}c_{5}c_{8}-576\beta^{2}c_{0}c_{6}c_{7}\\ \; & -440\beta^{2}c_{1}{}^{2}c_{11}-736\beta^{2}c_{1}c_{2}c_{10}-624\beta^{2}c_{1}c_{3}c_{9}-544\beta^{2}c_{1}c_{4}c_{8}-496\beta^{2}c_{1}c_{5}c_{7}\\ \; & -240\beta^{2}c_{1}c_{6}{}^{2}-304\beta^{2}c_{2}{}^{2}c_{9}-512\beta^{2}c_{2}c_{3}c_{8}-448\beta^{2}c_{2}c_{4}c_{7}-416\beta^{2}c_{2}c_{5}c_{6}\\ \; & -216\beta^{2}c_{3}{}^{2}c_{7}-384\beta^{2}c_{3}c_{4}c_{6}-184\beta^{2}c_{3}c_{5}{}^{2}-176\beta^{2}c_{4}{}^{2}c_{5}+450Q^{2}\beta c_{10}\\ \; & -1200\beta c_{0}{}^{2}c_{12}-2048\beta c_{0}c_{1}c_{11}-1760\beta c_{0}c_{2}c_{10}-1536\beta c_{0}c_{3}c_{9}-1376\beta c_{0}c_{4}c_{8}\\ \; & -1280\beta c_{0}c_{5}c_{7}-624\beta c_{0}c_{6}{}^{2}-864\beta c_{1}{}^{2}c_{10}-1472\beta c_{1}c_{2}c_{9}-1280\beta c_{1}c_{3}c_{8}\\ \; & -1152\beta c_{1}c_{4}c_{7}-1088\beta c_{1}c_{5}c_{6}-624\beta c_{2}{}^{2}c_{8}-1088\beta c_{2}c_{3}c_{7}-992\beta c_{2}c_{4}c_{6}\\ \; & -480\beta c_{2}c_{5}{}^{2}-480\beta c_{3}{}^{2}c_{6}-896\beta c_{3}c_{4}c_{5}-144\beta c_{4}{}^{3}+99Q^{2}c_{9}-572c_{0}{}^{2}c_{11}\\ \; & -984c_{0}c_{1}c_{10}-856c_{0}c_{2}c_{9}-760c_{0}c_{3}c_{8}-696c_{0}c_{4}c_{7}-664c_{0}c_{5}c_{6}-420c_{1}{}^{2}c_{9}\\ \; & -728c_{1}c_{2}c_{8}-648c_{1}c_{3}c_{7}-600c_{1}c_{4}c_{6}-292c_{1}c_{5}{}^{2}-316c_{2}{}^{2}c_{7}-568c_{2}c_{3}c_{6}\\ \; & \left.-536c_{2}c_{4}c_{5}-260c_{3}{}^{2}c_{5}-252c_{3}c_{4}{}^{2}\right) \end{array},$$

$$\begin{array}{cc} \; & c_{14}=-\frac{1}{182\beta^{2}\left(Q^{2}\beta^{2}-4c_{0}{}^{2}\right)}\left(663Q^{2}\beta^{3}c_{13}+900Q^{2}\beta^{2}c_{12}-1248\beta^{2}c_{0}c_{1}c_{13}\right.\\ \; & -1072\beta^{2}c_{0}c_{2}c_{12}-928\beta^{2}c_{0}c_{3}c_{11}-816\beta^{2}c_{0}c_{4}c_{10}-736\beta^{2}c_{0}c_{5}c_{9}-688\beta^{2}c_{0}c_{6}c_{8}\\ \; & -336\beta^{2}c_{0}c_{7}{}^{2}-528\beta^{2}c_{1}{}^{2}c_{12}-896\beta^{2}c_{1}c_{2}c_{11}-768\beta^{2}c_{1}c_{3}c_{10}-672\beta^{2}c_{1}c_{4}c_{9}\\ \; & -608\beta^{2}c_{1}c_{5}c_{8}-576\beta^{2}c_{1}c_{6}c_{7}-376\beta^{2}c_{2}{}^{2}c_{10}-640\beta^{2}c_{2}c_{3}c_{9}-560\beta^{2}c_{2}c_{4}c_{8}\\ \; & -512\beta^{2}c_{2}c_{5}c_{7}-248\beta^{2}c_{2}c_{6}{}^{2}-272\beta^{2}c_{3}{}^{2}c_{8}-480\beta^{2}c_{3}c_{4}c_{7}-448\beta^{2}c_{3}c_{5}c_{6}\\ \; & -216\beta^{2}c_{4}{}^{2}c_{6}-208\beta^{2}c_{4}c_{5}{}^{2}+539Q^{2}\beta c_{11}-1404\beta c_{0}{}^{2}c_{13}-2424\beta c_{0}c_{1}c_{12}\\ \; & -2104\beta c_{0}c_{2}c_{11}-1848\beta c_{0}c_{3}c_{10}-1656\beta c_{0}c_{4}c_{9}-1528\beta c_{0}c_{5}c_{8}-1464\beta c_{0}c_{6}c_{7}\\ \; & -1036\beta c_{1}{}^{2}c_{11}-1784\beta c_{1}c_{2}c_{10}-1560\beta c_{1}c_{3}c_{9}-1400\beta c_{1}c_{4}c_{8}-1304\beta c_{1}c_{5}c_{7}\\ \; & -636\beta c_{1}c_{6}{}^{2}-764\beta c_{2}{}^{2}c_{9}-1336\beta c_{2}c_{3}c_{8}-1208\beta c_{2}c_{4}c_{7}-1144\beta c_{2}c_{5}c_{6}\\ \; & -588\beta c_{3}{}^{2}c_{7}-1080\beta c_{3}c_{4}c_{6}-524\beta c_{3}c_{5}{}^{2}-508\beta c_{4}{}^{2}c_{5}+120Q^{2}c_{10}-672c_{0}{}^{2}c_{12}\\ \; & -1168c_{0}c_{1}c_{11}-1024c_{0}c_{2}c_{10}-912c_{0}c_{3}c_{9}-832c_{0}c_{4}c_{8}-784c_{0}c_{5}c_{7}-384c_{0}c_{6}{}^{2}\\ \; & -504c_{1}{}^{2}c_{10}-880c_{1}c_{2}c_{9}-784c_{1}c_{3}c_{8}-720c_{1}c_{4}c_{7}-688c_{1}c_{5}c_{6}-384c_{2}{}^{2}c_{8}\\ \; & \left.-688c_{2}c_{3}c_{7}-640c_{2}c_{4}c_{6}-312c_{2}c_{5}{}^{2}-312c_{3}{}^{2}c_{6}-592c_{3}c_{4}c_{5}-96c_{4}{}^{3}\right) \end{array},$$

$$\begin{array}{cc} \; & c_{15}=-\frac{1}{210\beta^{2}\left(Q^{2}\beta^{2}-4c_{0}{}^{2}\right)}\left(770Q^{2}\beta^{3}c_{14}+1053Q^{2}\beta^{2}c_{13}-1456\beta^{2}c_{0}c_{1}c_{14}\right.\\ \; & -1264\beta^{2}c_{0}c_{2}c_{13}-1104\beta^{2}c_{0}c_{3}c_{12}-976\beta^{2}c_{0}c_{4}c_{11}-880\beta^{2}c_{0}c_{5}c_{10}\\ \; & -816\beta^{2}c_{0}c_{6}c_{9}-784\beta^{2}c_{0}c_{7}c_{8}-624\beta^{2}c_{1}{}^{2}c_{13}-1072\beta^{2}c_{1}c_{2}c_{12}\\ \; & -928\beta^{2}c_{1}c_{3}c_{11}-816\beta^{2}c_{1}c_{4}c_{10}-736\beta^{2}c_{1}c_{5}c_{9}-688\beta^{2}c_{1}c_{6}c_{8}\\ \; & -336\beta^{2}c_{1}c_{7}{}^{2}-456\beta^{2}c_{2}{}^{2}c_{11}-784\beta^{2}c_{2}c_{3}c_{10}-688\beta^{2}c_{2}c_{4}c_{9}\\ \; & -624\beta^{2}c_{2}c_{5}c_{8}-592\beta^{2}c_{2}c_{6}c_{7}-336\beta^{2}c_{3}{}^{2}c_{9}-592\beta^{2}c_{3}c_{4}c_{8}\\ \; & -544\beta^{2}c_{3}c_{5}c_{7}-264\beta^{2}c_{3}c_{6}{}^{2}-264\beta^{2}c_{4}{}^{2}c_{7}-496\beta^{2}c_{4}c_{5}c_{6}-80\beta^{2}c_{5}{}^{3}\\ \; & +636Q^{2}\beta c_{12}-1624\beta c_{0}{}^{2}c_{14}-2832\beta c_{0}c_{1}c_{13}-2480\beta c_{0}c_{2}c_{12}\\ \; & -2192\beta c_{0}c_{3}c_{11}-1968\beta c_{0}c_{4}c_{10}-1808\beta c_{0}c_{5}c_{9}-1712\beta c_{0}c_{6}c_{8}\\ \; & -840\beta c_{0}c_{7}{}^{2}-1224\beta c_{1}{}^{2}c_{12}-2128\beta c_{1}c_{2}c_{11}-1872\beta c_{1}c_{3}c_{10}\\ \; & -1680\beta c_{1}c_{4}c_{9}-1552\beta c_{1}c_{5}c_{8}-1488\beta c_{1}c_{6}c_{7}-920\beta c_{2}{}^{2}c_{10}\\ \; & -1616\beta c_{2}c_{3}c_{9}-1456\beta c_{2}c_{4}c_{8}-1360\beta c_{2}c_{5}c_{7}-664\beta c_{2}c_{6}{}^{2}\\ \; & -712\beta c_{3}{}^{2}c_{8}-1296\beta c_{3}c_{4}c_{7}-1232\beta c_{3}c_{5}c_{6}-600\beta c_{4}{}^{2}c_{6}\\ \; & -584\beta c_{4}c_{5}{}^{2}+143Q^{2}c_{11}-780c_{0}{}^{2}c_{13}-1368c_{0}c_{1}c_{12}-1208c_{0}c_{2}c_{11}\\ \; & -1080c_{0}c_{3}c_{10}-984c_{0}c_{4}c_{9}-920c_{0}c_{5}c_{8}-888c_{0}c_{6}c_{7}-596c_{1}{}^{2}c_{11}\\ \; & -1048c_{1}c_{2}c_{10}-936c_{1}c_{3}c_{9}-856c_{1}c_{4}c_{8}-808c_{1}c_{5}c_{7}-396c_{1}c_{6}{}^{2}\\ \; & -460c_{2}{}^{2}c_{9}-824c_{2}c_{3}c_{8}-760c_{2}c_{4}c_{7}-728c_{2}c_{5}c_{6}-372c_{3}{}^{2}c_{7}\\ \; & \left.-696c_{3}c_{4}c_{6}-340c_{3}c_{5}{}^{2}-332c_{4}{}^{2}c_{5}\right) \end{array}.$$

## Appendix B

$$d_{1}=-\frac{\beta Q}{\sqrt{-Q^{2}\beta^{2}+4c_{0}{}^{2}}},$$

$$d_{2}=-\frac{Q^{2}\beta d_{1}{}^{2}-4c_{0}c_{1}d_{1}{}^{2}+Q^{2}\beta }{2d_{1}(Q^{2}\beta^{2}-4c_{0}{}^{2})},$$

$$\begin{array}{l} d_{3}=-\frac{1}{6d_{1}(Q^{2}\beta^{2}-4c_{0}{}^{2})}(4Q^{2}\beta^{2}d_{2}{}^{2}+8Q^{2}\beta d_{1}d_{2}+Q^{2}d_{1}{}^{2}-16c_{0}{}^{2}d_{2}{}^{2}-32c_{0}c_{1}d_{1}d_{2}\\ -8c_{0}c_{2}d_{1}{}^{2}-4c_{1}{}^{2}d_{1}{}^{2}+Q^{2}) \end{array},$$

$$\begin{array}{l} d_{4}=-\frac{1}{2d_{1}(Q^{2}\beta^{2}-4c_{0}{}^{2})}(3Q^{2}\beta^{2}d_{2}d_{3}+3Q^{2}\beta d_{1}d_{3}+2Q^{2}\beta d_{2}{}^{2}+Q^{2}d_{1}d_{2}\\ -12c_{0}{}^{2}d_{2}d_{3}-12c_{0}c_{1}d_{1}d_{3}-8c_{0}c_{1}d_{2}{}^{2}-8c_{0}c_{2}d_{1}d_{2}-2c_{0}c_{3}d_{1}{}^{2}-4c_{1}{}^{2}d_{1}d_{2}-2c_{1}c_{2}d_{1}{}^{2}) \end{array},$$

$$\begin{array}{l} d_{5}=-\frac{1}{10d_{1}(Q^{2}\beta^{2}-4c_{0}{}^{2})}(16Q^{2}\beta^{2}d_{2}d_{4}+9Q^{2}\beta^{2}d_{3}{}^{2}+16Q^{2}\beta d_{1}d_{4}+24Q^{2}\beta d_{2}d_{3}\\ +6Q^{2}d_{1}d_{3}+4Q^{2}d_{2}{}^{2}-64c_{0}{}^{2}d_{2}d_{4}-36c_{0}{}^{2}d_{3}{}^{2}-64c_{0}c_{1}d_{1}d_{4}-96c_{0}c_{1}d_{2}d_{3}-48c_{0}c_{2}d_{1}d_{3}\\ -32c_{0}c_{2}d_{2}{}^{2}-32c_{0}c_{3}d_{1}d_{2}-8c_{0}c_{4}d_{1}{}^{2}-24c_{1}{}^{2}d_{1}d_{3}-16c_{1}{}^{2}d_{2}{}^{2}-32c_{1}c_{2}d_{1}d_{2}-8c_{1}c_{3}d_{1}{}^{2}\\ -4c_{2}{}^{2}d_{1}{}^{2}) \end{array},$$

$$\begin{array}{cc} \; & d_{6}=-\frac{1}{6d_{1}\left(Q^{2}\beta^{2}-4c_{0}{}^{2}\right)}\left(10Q^{2}\beta^{2}d_{2}d_{5}+12Q^{2}\beta^{2}d_{3}d_{4}+10Q^{2}\beta d_{1}d_{5}+16Q^{2}\beta d_{2}d_{4}\right.\\ \; & +9Q^{2}\beta d_{3}{}^{2}+4Q^{2}d_{1}d_{4}+6Q^{2}d_{2}d_{3}-40c_{0}{}^{2}d_{2}d_{5}-48c_{0}{}^{2}d_{3}d_{4}-40c_{0}c_{1}d_{1}d_{5}\\ \; & -64c_{0}c_{1}d_{2}d_{4}-36c_{0}c_{1}d_{3}{}^{2}-32c_{0}c_{2}d_{1}d_{4}-48c_{0}c_{2}d_{2}d_{3}-24c_{0}c_{3}d_{1}d_{3}-16c_{0}c_{3}d_{2}{}^{2}\\ \; & -16c_{0}c_{4}d_{1}d_{2}-4c_{0}c_{5}d_{1}{}^{2}-16c_{1}{}^{2}d_{1}d_{4}-24c_{1}{}^{2}d_{2}d_{3}-24c_{1}c_{2}d_{1}d_{3}-16c_{1}c_{2}d_{2}{}^{2}\\ \; & \left.-16c_{1}c_{3}d_{1}d_{2}-4c_{1}c_{4}d_{1}{}^{2}-8c_{2}{}^{2}d_{1}d_{2}-4c_{2}c_{3}d_{1}{}^{2}\right) \end{array},$$

$$\begin{array}{cc} \; & d_{7}=-\frac{1}{14d_{1}\left(Q^{2}\beta^{2}-4c_{0}{}^{2}\right)}\left(24Q^{2}\beta^{2}d_{2}d_{6}+30Q^{2}\beta^{2}d_{3}d_{5}+16Q^{2}\beta^{2}d_{4}{}^{2}+24Q^{2}\beta d_{1}d_{6}\right.\\ \; & +40Q^{2}\beta d_{2}d_{5}+48Q^{2}\beta d_{3}d_{4}+10Q^{2}d_{1}d_{5}+16Q^{2}d_{2}d_{4}+9Q^{2}d_{3}{}^{2}-96c_{0}{}^{2}d_{2}d_{6}\\ \; & -120c_{0}{}^{2}d_{3}d_{5}-64c_{0}{}^{2}d_{4}{}^{2}-96c_{0}c_{1}d_{1}d_{6}-160c_{0}c_{1}d_{2}d_{5}-192c_{0}c_{1}d_{3}d_{4}-80c_{0}c_{2}d_{1}d_{5}\\ \; & -128c_{0}c_{2}d_{2}d_{4}-72c_{0}c_{2}d_{3}{}^{2}-64c_{0}c_{3}d_{1}d_{4}-96c_{0}c_{3}d_{2}d_{3}-48c_{0}c_{4}d_{1}d_{3}-32c_{0}c_{4}d_{2}{}^{2}\\ \; & -32c_{0}c_{5}d_{1}d_{2}-8c_{0}c_{6}d_{1}{}^{2}-40c_{1}{}^{2}d_{1}d_{5}-64c_{1}{}^{2}d_{2}d_{4}-36c_{1}{}^{2}d_{3}{}^{2}-64c_{1}c_{2}d_{1}d_{4}\\ \; & -96c_{1}c_{2}d_{2}d_{3}-48c_{1}c_{3}d_{1}d_{3}-32c_{1}c_{3}d_{2}{}^{2}-32c_{1}c_{4}d_{1}d_{2}-8c_{1}c_{5}d_{1}{}^{2}-24c_{2}{}^{2}d_{1}d_{3}\\ \; & \left.-16c_{2}{}^{2}d_{2}{}^{2}-32c_{2}c_{3}d_{1}d_{2}-8c_{2}c_{4}d_{1}{}^{2}-4c_{3}{}^{2}d_{1}{}^{2}\right) \end{array},$$

$$\begin{array}{cc} \; & d_{8}=-\frac{1}{4d_{1}\left(Q^{2}\beta^{2}-4c_{0}{}^{2}\right)}\left(7Q^{2}\beta^{2}d_{2}d_{7}+9Q^{2}\beta^{2}d_{3}d_{6}+10Q^{2}\beta^{2}d_{4}d_{5}+7Q^{2}\beta d_{1}d_{7}\right.\\ \; & +12Q^{2}\beta d_{2}d_{6}+15Q^{2}\beta d_{3}d_{5}+8Q^{2}\beta d_{4}{}^{2}+3Q^{2}d_{1}d_{6}+5Q^{2}d_{2}d_{5}+6Q^{2}d_{3}d_{4}\\ \; & -28c_{0}{}^{2}d_{2}d_{7}-36c_{0}{}^{2}d_{3}d_{6}-40c_{0}{}^{2}d_{4}d_{5}-28c_{0}c_{1}d_{1}d_{7}-48c_{0}c_{1}d_{2}d_{6}-60c_{0}c_{1}d_{3}d_{5}\\ \; & -32c_{0}c_{1}d_{4}{}^{2}-24c_{0}c_{2}d_{1}d_{6}-40c_{0}c_{2}d_{2}d_{5}-48c_{0}c_{2}d_{3}d_{4}-20c_{0}c_{3}d_{1}d_{5}-32c_{0}c_{3}d_{2}d_{4}\\ \; & -18c_{0}c_{3}d_{3}{}^{2}-16c_{0}c_{4}d_{1}d_{4}-24c_{0}c_{4}d_{2}d_{3}-12c_{0}c_{5}d_{1}d_{3}-8c_{0}c_{5}d_{2}{}^{2}-8c_{0}c_{6}d_{1}d_{2}\\ \; & -2c_{0}c_{7}d_{1}{}^{2}-12c_{1}{}^{2}d_{1}d_{6}-20c_{1}{}^{2}d_{2}d_{5}-24c_{1}{}^{2}d_{3}d_{4}-20c_{1}c_{2}d_{1}d_{5}-32c_{1}c_{2}d_{2}d_{4}\\ \; & -18c_{1}c_{2}d_{3}{}^{2}-16c_{1}c_{3}d_{1}d_{4}-24c_{1}c_{3}d_{2}d_{3}-12c_{1}c_{4}d_{1}d_{3}-8c_{1}c_{4}d_{2}{}^{2}-8c_{1}c_{5}d_{1}d_{2}\\ \; & -2c_{1}c_{6}d_{1}{}^{2}-8c_{2}{}^{2}d_{1}d_{4}-12c_{2}{}^{2}d_{2}d_{3}-12c_{2}c_{3}d_{1}d_{3}-8c_{2}c_{3}d_{2}{}^{2}-8c_{2}c_{4}d_{1}d_{2}-2c_{2}c_{5}d_{1}{}^{2}\\ \; & \left.-4c_{3}{}^{2}d_{1}d_{2}-2c_{3}c_{4}d_{1}{}^{2}\right) \end{array},$$

$$\begin{array}{cc} \; & d_{9}=-\frac{1}{18d_{1}\left(Q^{2}\beta^{2}-4c_{0}{}^{2}\right)}\left(32Q^{2}\beta^{2}d_{2}d_{8}+42Q^{2}\beta^{2}d_{3}d_{7}+48Q^{2}\beta^{2}d_{4}d_{6}\right.\\ \; & +25Q^{2}\beta^{2}d_{5}{}^{2}+32Q^{2}\beta d_{1}d_{8}+56Q^{2}\beta d_{2}d_{7}+72Q^{2}\beta d_{3}d_{6}+80Q^{2}\beta d_{4}d_{5}\\ \; & +14Q^{2}d_{1}d_{7}+24Q^{2}d_{2}d_{6}+30Q^{2}d_{3}d_{5}+16Q^{2}d_{4}{}^{2}-128c_{0}{}^{2}d_{2}d_{8}-168c_{0}{}^{2}d_{3}d_{7}\\ \; & -192c_{0}{}^{2}d_{4}d_{6}-100c_{0}{}^{2}d_{5}{}^{2}-128c_{0}c_{1}d_{1}d_{8}-224c_{0}c_{1}d_{2}d_{7}-288c_{0}c_{1}d_{3}d_{6}\\ \; & -320c_{0}c_{1}d_{4}d_{5}-112c_{0}c_{2}d_{1}d_{7}-192c_{0}c_{2}d_{2}d_{6}-240c_{0}c_{2}d_{3}d_{5}-128c_{0}c_{2}d_{4}{}^{2}\\ \; & -96c_{0}c_{3}d_{1}d_{6}-160c_{0}c_{3}d_{2}d_{5}-192c_{0}c_{3}d_{3}d_{4}-80c_{0}c_{4}d_{1}d_{5}-128c_{0}c_{4}d_{2}d_{4}\\ \; & -72c_{0}c_{4}d_{3}{}^{2}-64c_{0}c_{5}d_{1}d_{4}-96c_{0}c_{5}d_{2}d_{3}-48c_{0}c_{6}d_{1}d_{3}-32c_{0}c_{6}d_{2}{}^{2}\\ \; & -32c_{0}c_{7}d_{1}d_{2}-8c_{0}c_{8}d_{1}{}^{2}-56c_{1}{}^{2}d_{1}d_{7}-96c_{1}{}^{2}d_{2}d_{6}-120c_{1}{}^{2}d_{3}d_{5}-64c_{1}{}^{2}d_{4}{}^{2}\\ \; & -96c_{1}c_{2}d_{1}d_{6}-160c_{1}c_{2}d_{2}d_{5}-192c_{1}c_{2}d_{3}d_{4}-80c_{1}c_{3}d_{1}d_{5}-128c_{1}c_{3}d_{2}d_{4}\\ \; & -72c_{1}c_{3}d_{3}{}^{2}-64c_{1}c_{4}d_{1}d_{4}-96c_{1}c_{4}d_{2}d_{3}-48c_{1}c_{5}d_{1}d_{3}-32c_{1}c_{5}d_{2}{}^{2}-32c_{1}c_{6}d_{1}d_{2}\\ \; & -8c_{1}c_{7}d_{1}{}^{2}-40c_{2}{}^{2}d_{1}d_{5}-64c_{2}{}^{2}d_{2}d_{4}-36c_{2}{}^{2}d_{3}{}^{2}-64c_{2}c_{3}d_{1}d_{4}-96c_{2}c_{3}d_{2}d_{3}\\ \; & -48c_{2}c_{4}d_{1}d_{3}-32c_{2}c_{4}d_{2}{}^{2}-32c_{2}c_{5}d_{1}d_{2}-8c_{2}c_{6}d_{1}{}^{2}-24c_{3}{}^{2}d_{1}d_{3}-16c_{3}{}^{2}d_{2}{}^{2}\\ \; & \left.-32c_{3}c_{4}d_{1}d_{2}-8c_{3}c_{5}d_{1}{}^{2}-4c_{4}{}^{2}d_{1}{}^{2}\right) \end{array},$$

$$\begin{array}{cc} \; & d_{10}=-\frac{1}{10d_{1}\left(Q^{2}\beta^{2}-4c_{0}{}^{2}\right)}\left(18Q^{2}\beta^{2}d_{2}d_{9}+24Q^{2}\beta^{2}d_{3}d_{8}+28Q^{2}\beta^{2}d_{4}d_{7}\right.\\ \; & +30Q^{2}\beta^{2}d_{5}d_{6}+18Q^{2}\beta d_{1}d_{9}+32Q^{2}\beta d_{2}d_{8}+42Q^{2}\beta d_{3}d_{7}+48Q^{2}\beta d_{4}d_{6}\\ \; & +25Q^{2}\beta d_{5}{}^{2}+8Q^{2}d_{1}d_{8}+14Q^{2}d_{2}d_{7}+18Q^{2}d_{3}d_{6}+20Q^{2}d_{4}d_{5}-72c_{0}{}^{2}d_{2}d_{9}\\ \; & -96c_{0}{}^{2}d_{3}d_{8}-112c_{0}{}^{2}d_{4}d_{7}-120c_{0}{}^{2}d_{5}d_{6}-72c_{0}c_{1}d_{1}d_{9}-128c_{0}c_{1}d_{2}d_{8}\\ \; & -168c_{0}c_{1}d_{3}d_{7}-192c_{0}c_{1}d_{4}d_{6}-100c_{0}c_{1}d_{5}{}^{2}-64c_{0}c_{2}d_{1}d_{8}-112c_{0}c_{2}d_{2}d_{7}\\ \; & -144c_{0}c_{2}d_{3}d_{6}-160c_{0}c_{2}d_{4}d_{5}-56c_{0}c_{3}d_{1}d_{7}-96c_{0}c_{3}d_{2}d_{6}-120c_{0}c_{3}d_{3}d_{5}\\ \; & -64c_{0}c_{3}d_{4}{}^{2}-48c_{0}c_{4}d_{1}d_{6}-80c_{0}c_{4}d_{2}d_{5}-96c_{0}c_{4}d_{3}d_{4}-40c_{0}c_{5}d_{1}d_{5}\\ \; & -64c_{0}c_{5}d_{2}d_{4}-36c_{0}c_{5}d_{3}{}^{2}-32c_{0}c_{6}d_{1}d_{4}-48c_{0}c_{6}d_{2}d_{3}-24c_{0}c_{7}d_{1}d_{3}\\ \; & -16c_{0}c_{7}d_{2}{}^{2}-16c_{0}c_{8}d_{1}d_{2}-4c_{0}c_{9}d_{1}{}^{2}-32c_{1}{}^{2}d_{1}d_{8}-56c_{1}{}^{2}d_{2}d_{7}-72c_{1}d_{3}d_{6}\\ \; & -80c_{1}{}^{2}d_{4}d_{5}-56c_{1}c_{2}d_{1}d_{7}-96c_{1}c_{2}d_{2}d_{6}-120c_{1}c_{2}d_{3}d_{5}-64c_{1}c_{2}d_{4}{}^{2}-48c_{1}c_{3}d_{1}d_{6}\\ \; & -80c_{1}c_{3}d_{2}d_{5}-96c_{1}c_{3}d_{3}d_{4}-40c_{1}c_{4}d_{1}d_{5}-64c_{1}c_{4}d_{2}d_{4}-36c_{1}c_{4}d_{3}{}^{2}-32c_{1}c_{5}d_{1}d_{4}\\ \; & -48c_{1}c_{5}d_{2}d_{3}-24c_{1}c_{6}d_{1}d_{3}-16c_{1}c_{6}d_{2}{}^{2}-16c_{1}c_{7}d_{1}d_{2}-4c_{1}c_{8}d_{1}{}^{2}-24c_{2}{}^{2}d_{1}d_{6}\\ \; & -40c_{2}{}^{2}d_{2}d_{5}-48c_{2}{}^{2}d_{3}d_{4}-40c_{2}c_{3}d_{1}d_{5}-64c_{2}c_{3}d_{2}d_{4}-36c_{2}c_{3}d_{3}{}^{2}-32c_{2}c_{4}d_{1}d_{4}\\ \; & -48c_{2}c_{4}d_{2}d_{3}-24c_{2}c_{5}d_{1}d_{3}-16c_{2}c_{5}d_{2}{}^{2}-16c_{2}c_{6}d_{1}d_{2}-4c_{2}c_{7}d_{1}{}^{2}-16c_{3}{}^{2}d_{1}d_{4}\\ \; & -24c_{3}{}^{2}d_{2}d_{3}-24c_{3}c_{4}d_{1}d_{3}-16c_{3}c_{4}d_{2}{}^{2}-16c_{3}c_{5}d_{1}d_{2}-4c_{3}c_{6}d_{1}{}^{2}-8c_{4}{}^{2}d_{1}d_{2}\\ \; & \left.-4c_{4}c_{5}d_{1}{}^{2}\right) \end{array},$$

$$\begin{array}{cc} \; & d_{11}=-\frac{1}{22d_{1}\left(Q^{2}\beta^{2}-4c_{0}{}^{2}\right)}\left(40Q^{2}\beta^{2}d_{2}d_{10}+54Q^{2}\beta^{2}d_{3}d_{9}+64Q^{2}\beta^{2}d_{4}d_{8}\right.\\ \; & +70Q^{2}\beta^{2}d_{5}d_{7}+36Q^{2}\beta^{2}d_{6}{}^{2}+40Q^{2}\beta d_{1}d_{10}+72Q^{2}\beta d_{2}d_{9}+96Q^{2}\beta d_{3}d_{8}\\ \; & +112Q^{2}\beta d_{4}d_{7}+120Q^{2}\beta d_{5}d_{6}+18Q^{2}d_{1}d_{9}+32Q^{2}d_{2}d_{8}+42Q^{2}d_{3}d_{7}\\ \; & +48Q^{2}d_{4}d_{6}+25Q^{2}d_{5}{}^{2}-160c_{0}{}^{2}d_{2}d_{10}-216c_{0}{}^{2}d_{3}d_{9}-256c_{0}{}^{2}d_{4}d_{8}-280c_{0}{}^{2}d_{5}d_{7}\\ \; & -144c_{0}{}^{2}d_{6}{}^{2}-160c_{0}c_{1}d_{1}d_{10}-288c_{0}c_{1}d_{2}d_{9}-384c_{0}c_{1}d_{3}d_{8}-448c_{0}c_{1}d_{4}d_{7}\\ \; & -480c_{0}c_{1}d_{5}d_{6}-144c_{0}c_{2}d_{1}d_{9}-256c_{0}c_{2}d_{2}d_{8}-336c_{0}c_{2}d_{3}d_{7}-384c_{0}c_{2}d_{4}d_{6}\\ \; & -200c_{0}c_{2}d_{5}{}^{2}-128c_{0}c_{3}d_{1}d_{8}-224c_{0}c_{3}d_{2}d_{7}-288c_{0}c_{3}d_{3}d_{6}-320c_{0}c_{3}d_{4}d_{5}\\ \; & -112c_{0}c_{4}d_{1}d_{7}-192c_{0}c_{4}d_{2}d_{6}-240c_{0}c_{4}d_{3}d_{5}-128c_{0}c_{4}d_{4}{}^{2}-96c_{0}c_{5}d_{1}d_{6}\\ \; & -160c_{0}c_{5}d_{2}d_{5}-192c_{0}c_{5}d_{3}d_{4}-80c_{0}c_{6}d_{1}d_{5}-128c_{0}c_{6}d_{2}d_{4}-72c_{0}c_{6}d_{3}{}^{2}\\ \; & -64c_{0}c_{7}d_{1}d_{4}-96c_{0}c_{7}d_{2}d_{3}-48c_{0}c_{8}d_{1}d_{3}-32c_{0}c_{8}d_{2}{}^{2}-32c_{0}c_{9}d_{1}d_{2}\\ \; & -8c_{0}c_{10}d_{1}{}^{2}-72c_{1}{}^{2}d_{1}d_{9}-128c_{1}{}^{2}d_{2}d_{8}-168c_{1}{}^{2}d_{3}d_{7}-192c_{1}{}^{2}d_{4}d_{6}-100c_{1}{}^{2}d_{5}{}^{2}\\ \; & -128c_{1}c_{2}d_{1}d_{8}-224c_{1}c_{2}d_{2}d_{7}-288c_{1}c_{2}d_{3}d_{6}-320c_{1}c_{2}d_{4}d_{5}-112c_{1}c_{3}d_{1}d_{7}\\ \; & -192c_{1}c_{3}d_{2}d_{6}-240c_{1}c_{3}d_{3}d_{5}-128c_{1}c_{3}d_{4}{}^{2}-96c_{1}c_{4}d_{1}d_{6}-160c_{1}c_{4}d_{2}d_{5}\\ \; & -192c_{1}c_{4}d_{3}d_{4}-80c_{1}c_{5}d_{1}d_{5}-128c_{1}c_{5}d_{2}d_{4}-72c_{1}c_{5}d_{3}{}^{2}-64c_{1}c_{6}d_{1}d_{4}\\ \; & -96c_{1}c_{6}d_{2}d_{3}-48c_{1}c_{7}d_{1}d_{3}-32c_{1}c_{7}d_{2}{}^{2}-32c_{1}c_{8}d_{1}d_{2}-8c_{1}c_{9}d_{1}{}^{2}-56c_{2}{}^{2}d_{1}d_{7}\\ \; & -96c_{2}{}^{2}d_{2}d_{6}-120c_{2}{}^{2}d_{3}d_{5}-64c_{2}{}^{2}d_{4}{}^{2}-96c_{2}c_{3}d_{1}d_{6}-160c_{2}c_{3}d_{2}d_{5}-192c_{2}c_{3}d_{3}d_{4}\\ \; & -80c_{2}c_{4}d_{1}d_{5}-128c_{2}c_{4}d_{2}d_{4}-72c_{2}c_{4}d_{3}{}^{2}-64c_{2}c_{5}d_{1}d_{4}-96c_{2}c_{5}d_{2}d_{3}\\ \; & -48c_{2}c_{6}d_{1}d_{3}-32c_{2}c_{6}d_{2}{}^{2}-32c_{2}c_{7}d_{1}d_{2}-8c_{2}c_{8}d_{1}{}^{2}-40c_{3}{}^{2}d_{1}d_{5}-64c_{3}{}^{2}d_{2}d_{4}\\ \; & -36c_{3}{}^{2}d_{3}{}^{2}-64c_{3}c_{4}d_{1}d_{4}-96c_{3}c_{4}d_{2}d_{3}-48c_{3}c_{5}d_{1}d_{3}-32c_{3}c_{5}d_{2}{}^{2}-32c_{3}c_{6}d_{1}d_{2}\\ \; & \left.-8c_{3}c_{7}d_{1}{}^{2}-24c_{4}{}^{2}d_{1}d_{3}-16c_{4}{}^{2}d_{2}{}^{2}-32c_{4}c_{5}d_{1}d_{2}-8c_{4}c_{6}d_{1}{}^{2}-4c_{5}{}^{2}d_{1}{}^{2}\right) \end{array},$$

$$\begin{array}{cc} \; & d_{12}=-\frac{1}{6d_{1}\left(Q^{2}\beta^{2}-4c_{0}{}^{2}\right)}\left(11Q^{2}\beta^{2}d_{2}d_{11}+15Q^{2}\beta^{2}d_{3}d_{10}+18Q^{2}\beta^{2}d_{4}d_{9}\right.\\ \; & +20Q^{2}\beta^{2}d_{5}d_{8}+21Q^{2}\beta^{2}d_{6}d_{7}+11Q^{2}\beta d_{1}d_{11}+20Q^{2}\beta d_{2}d_{10}+27Q^{2}\beta d_{3}d_{9}\\ \; & +32Q^{2}\beta d_{4}d_{8}+35Q^{2}\beta d_{5}d_{7}+18Q^{2}\beta d_{6}{}^{2}+5Q^{2}d_{1}d_{10}+9Q^{2}d_{2}d_{9}\\ \; & +12Q^{2}d_{3}d_{8}+14Q^{2}d_{4}d_{7}+15Q^{2}d_{5}d_{6}-44c_{0}{}^{2}d_{2}d_{11}-60c_{0}{}^{2}d_{3}d_{10}\\ \; & -72c_{0}{}^{2}d_{4}d_{9}-80c_{0}{}^{2}d_{5}d_{8}-84c_{0}{}^{2}d_{6}d_{7}-44c_{0}c_{1}d_{1}d_{11}-80c_{0}c_{1}d_{2}d_{10}\\ \; & -108c_{0}c_{1}d_{3}d_{9}-128c_{0}c_{1}d_{4}d_{8}-140c_{0}c_{1}d_{5}d_{7}-72c_{0}c_{1}d_{6}{}^{2}-40c_{0}c_{2}d_{1}d_{10}\\ \; & -72c_{0}c_{2}d_{2}d_{9}-96c_{0}c_{2}d_{3}d_{8}-112c_{0}c_{2}d_{4}d_{7}-120c_{0}c_{2}d_{5}d_{6}-36c_{0}c_{3}d_{1}d_{9}\\ \; & -64c_{0}c_{3}d_{2}d_{8}-84c_{0}c_{3}d_{3}d_{7}-96c_{0}c_{3}d_{4}d_{6}-50c_{0}c_{3}d_{5}{}^{2}-32c_{0}c_{4}d_{1}d_{8}\\ \; & -56c_{0}c_{4}d_{2}d_{7}-72c_{0}c_{4}d_{3}d_{6}-80c_{0}c_{4}d_{4}d_{5}-28c_{0}c_{5}d_{1}d_{7}-48c_{0}c_{5}d_{2}d_{6}\\ \; & -60c_{0}c_{5}d_{3}d_{5}-32c_{0}c_{5}d_{4}{}^{2}-24c_{0}c_{6}d_{1}d_{6}-40c_{0}c_{6}d_{2}d_{5}-48c_{0}c_{6}d_{3}d_{4}\\ \; & -20c_{0}c_{7}d_{1}d_{5}-32c_{0}c_{7}d_{2}d_{4}-18c_{0}c_{7}d_{3}{}^{2}-16c_{0}c_{8}d_{1}d_{4}-24c_{0}c_{8}d_{2}d_{3}\\ \; & -12c_{0}c_{9}d_{1}d_{3}-8c_{0}c_{9}d_{2}{}^{2}-8c_{0}c_{10}d_{1}d_{2}-2c_{0}c_{11}d_{1}{}^{2}-20c_{1}{}^{2}d_{1}d_{10}\\ \; & -36c_{1}{}^{2}d_{2}d_{9}-48c_{1}{}^{2}d_{3}d_{8}-56c_{1}{}^{2}d_{4}d_{7}-60c_{1}{}^{2}d_{5}d_{6}-36c_{1}c_{2}d_{1}d_{9}\\ \; & -64c_{1}c_{2}d_{2}d_{8}-84c_{1}c_{2}d_{3}d_{7}-96c_{1}c_{2}d_{4}d_{6}-50c_{1}c_{2}d_{5}{}^{2}-32c_{1}c_{3}d_{1}d_{8}\\ \; & -56c_{1}c_{3}d_{2}d_{7}-72c_{1}c_{3}d_{3}d_{6}-80c_{1}c_{3}d_{4}d_{5}-28c_{1}c_{4}d_{1}d_{7}-48c_{1}c_{4}d_{2}d_{6}\\ \; & -60c_{1}c_{4}d_{3}d_{5}-32c_{1}c_{4}d_{4}{}^{2}-24c_{1}c_{5}d_{1}d_{6}-40c_{1}c_{5}d_{2}d_{5}-48c_{1}c_{5}d_{3}d_{4}\\ \; & -20c_{1}c_{6}d_{1}d_{5}-32c_{1}c_{6}d_{2}d_{4}-18c_{1}c_{6}d_{3}{}^{2}-16c_{1}c_{7}d_{1}d_{4}-24c_{1}c_{7}d_{2}d_{3}\\ \; & -12c_{1}c_{8}d_{1}d_{3}-8c_{1}c_{8}d_{2}{}^{2}-8c_{1}c_{9}d_{1}d_{2}-2c_{1}c_{10}d_{1}{}^{2}-16c_{2}{}^{2}d_{1}d_{8}\\ \; & -28c_{2}{}^{2}d_{2}d_{7}-36c_{2}{}^{2}d_{3}d_{6}-40c_{2}{}^{2}d_{4}d_{5}-28c_{2}c_{3}d_{1}d_{7}-48c_{2}c_{3}d_{2}d_{6}\\ \; & -60c_{2}c_{3}d_{3}d_{5}-32c_{2}c_{3}d_{4}{}^{2}-24c_{2}c_{4}d_{1}d_{6}-40c_{2}c_{4}d_{2}d_{5}-48c_{2}c_{4}d_{3}d_{4}\\ \; & -20c_{2}c_{5}d_{1}d_{5}-32c_{2}c_{5}d_{2}d_{4}-18c_{2}c_{5}d_{3}{}^{2}-16c_{2}c_{6}d_{1}d_{4}-24c_{2}c_{6}d_{2}d_{3}\\ \; & -12c_{2}c_{7}d_{1}d_{3}-8c_{2}c_{7}d_{2}{}^{2}-8c_{2}c_{8}d_{1}d_{2}-2c_{2}c_{9}d_{1}{}^{2}-12c_{3}{}^{2}d_{1}d_{6}\\ \; & -20c_{3}{}^{2}d_{2}d_{5}-24c_{3}{}^{2}d_{3}d_{4}-20c_{3}c_{4}d_{1}d_{5}-32c_{3}c_{4}d_{2}d_{4}-18c_{3}c_{4}d_{3}{}^{2}\\ \; & -16c_{3}c_{5}d_{1}d_{4}-24c_{3}c_{5}d_{2}d_{3}-12c_{3}c_{6}d_{1}d_{3}-8c_{3}c_{6}d_{2}{}^{2}-8c_{3}c_{7}d_{1}d_{2}\\ \; & -2c_{3}c_{8}d_{1}{}^{2}-8c_{4}{}^{2}d_{1}d_{4}-12c_{4}{}^{2}d_{2}d_{3}-12c_{4}c_{5}d_{1}d_{3}-8c_{4}c_{5}d_{2}{}^{2}\\ \; & \left.-8c_{4}c_{6}d_{1}d_{2}-2c_{4}c_{7}d_{1}{}^{2}-4c_{5}{}^{2}d_{1}d_{2}-2c_{5}c_{6}d_{1}{}^{2}\right) \end{array},$$

$$\begin{array}{cc} \; & d_{13}=-\frac{1}{26d_{1}\left(Q^{2}\beta^{2}-4c_{0}{}^{2}\right)}\left(48Q^{2}\beta^{2}d_{2}d_{12}+66Q^{2}\beta^{2}d_{3}d_{11}+80Q^{2}\beta^{2}d_{4}d_{10}\right.\\ \; & +90Q^{2}\beta^{2}d_{5}d_{9}+96Q^{2}\beta^{2}d_{6}d_{8}+49Q^{2}\beta^{2}d_{7}{}^{2}+48Q^{2}\beta d_{1}d_{12}+88Q^{2}\beta d_{2}d_{11}\\ \; & +120Q^{2}\beta d_{3}d_{10}+144Q^{2}\beta d_{4}d_{9}+160Q^{2}\beta d_{5}d_{8}+168Q^{2}\beta d_{6}d_{7}\\ \; & +22Q^{2}d_{1}d_{11}+40Q^{2}d_{2}d_{10}+54Q^{2}d_{3}d_{9}+64Q^{2}d_{4}d_{8}+70Q^{2}d_{5}d_{7}+36Q^{2}d_{6}{}^{2}\\ \; & -192c_{0}{}^{2}d_{2}d_{12}-264c_{0}{}^{2}d_{3}d_{11}-320c_{0}{}^{2}d_{4}d_{10}-360c_{0}{}^{2}d_{5}d_{9}-384c_{0}{}^{2}d_{6}d_{8}\\ \; & -196c_{0}{}^{2}d_{7}{}^{2}-192c_{0}c_{1}d_{1}d_{12}-352c_{0}c_{1}d_{2}d_{11}-480c_{0}c_{1}d_{3}d_{10}-576c_{0}c_{1}d_{4}d_{9}\\ \; & -640c_{0}c_{1}d_{5}d_{8}-672c_{0}c_{1}d_{6}d_{7}-176c_{0}c_{2}d_{1}d_{11}-320c_{0}c_{2}d_{2}d_{10}-432c_{0}c_{2}d_{3}d_{9}\\ \; & -512c_{0}c_{2}d_{4}d_{8}-560c_{0}c_{2}d_{5}d_{7}-288c_{0}c_{2}d_{6}{}^{2}-160c_{0}c_{3}d_{1}d_{10}-288c_{0}c_{3}d_{2}d_{9}\\ \; & -384c_{0}c_{3}d_{3}d_{8}-448c_{0}c_{3}d_{4}d_{7}-480c_{0}c_{3}d_{5}d_{6}-144c_{0}c_{4}d_{1}d_{9}-256c_{0}c_{4}d_{2}d_{8}\\ \; & -336c_{0}c_{4}d_{3}d_{7}-384c_{0}c_{4}d_{4}d_{6}-200c_{0}c_{4}d_{5}{}^{2}-128c_{0}c_{5}d_{1}d_{8}-224c_{0}c_{5}d_{2}d_{7}\\ \; & -288c_{0}c_{5}d_{3}d_{6}-320c_{0}c_{5}d_{4}d_{5}-112c_{0}c_{6}d_{1}d_{7}-192c_{0}c_{6}d_{2}d_{6}-240c_{0}c_{6}d_{3}d_{5}\\ \; & -128c_{0}c_{6}d_{4}{}^{2}-96c_{0}c_{7}d_{1}d_{6}-160c_{0}c_{7}d_{2}d_{5}-192c_{0}c_{7}d_{3}d_{4}-80c_{0}c_{8}d_{1}d_{5}\\ \; & -128c_{0}c_{8}d_{2}d_{4}-72c_{0}c_{8}d_{3}{}^{2}-64c_{0}c_{9}d_{1}d_{4}-96c_{0}c_{9}d_{2}d_{3}-48c_{0}c_{10}d_{1}d_{3}\\ \; & -32c_{0}c_{10}d_{2}{}^{2}-32c_{0}c_{11}d_{1}d_{2}-8c_{0}c_{12}d_{1}{}^{2}-88c_{1}{}^{2}d_{1}d_{11}-160c_{1}{}^{2}d_{2}d_{10}-216c_{1}{}^{2}d_{3}d_{9}\\ \; & -256c_{1}{}^{2}d_{4}d_{8}-280c_{1}{}^{2}d_{5}d_{7}-144c_{1}{}^{2}d_{6}{}^{2}-160c_{1}c_{2}d_{1}d_{10}-288c_{1}c_{2}d_{2}d_{9}\\ \; & -384c_{1}c_{2}d_{3}d_{8}-448c_{1}c_{2}d_{4}d_{7}-480c_{1}c_{2}d_{5}d_{6}-144c_{1}c_{3}d_{1}d_{9}-256c_{1}c_{3}d_{2}d_{8}\\ \; & -336c_{1}c_{3}d_{3}d_{7}-384c_{1}c_{3}d_{4}d_{6}-200c_{1}c_{3}d_{5}{}^{2}-128c_{1}c_{4}d_{1}d_{8}-224c_{1}c_{4}d_{2}d_{7}\\ \; & -288c_{1}c_{4}d_{3}d_{6}-320c_{1}c_{4}d_{4}d_{5}-112c_{1}c_{5}d_{1}d_{7}-192c_{1}c_{5}d_{2}d_{6}-240c_{1}c_{5}d_{3}d_{5}\\ \; & -128c_{1}c_{5}d_{4}{}^{2}-96c_{1}c_{6}d_{1}d_{6}-160c_{1}c_{6}d_{2}d_{5}-192c_{1}c_{6}d_{3}d_{4}-80c_{1}c_{7}d_{1}d_{5}\\ \; & -128c_{1}c_{7}d_{2}d_{4}-72c_{1}c_{7}d_{3}{}^{2}-64c_{1}c_{8}d_{1}d_{4}-96c_{1}c_{8}d_{2}d_{3}-48c_{1}c_{9}d_{1}d_{3}-32c_{1}c_{9}d_{2}{}^{2}\\ \; & -32c_{1}c_{10}d_{1}d_{2}-8c_{1}c_{11}d_{1}{}^{2}-72c_{2}{}^{2}d_{1}d_{9}-128c_{2}{}^{2}d_{2}d_{8}-168c_{2}{}^{2}d_{3}d_{7}-192c_{2}{}^{2}d_{4}d_{6}\\ \; & -100c_{2}{}^{2}d_{5}{}^{2}-128c_{2}c_{3}d_{1}d_{8}-224c_{2}c_{3}d_{2}d_{7}-288c_{2}c_{3}d_{3}d_{6}-320c_{2}c_{3}d_{4}d_{5}\\ \; & -112c_{2}c_{4}d_{1}d_{7}-192c_{2}c_{4}d_{2}d_{6}-240c_{2}c_{4}d_{3}d_{5}-128c_{2}c_{4}d_{4}{}^{2}-96c_{2}c_{5}d_{1}d_{6}\\ \; & -160c_{2}c_{5}d_{2}d_{5}-192c_{2}c_{5}d_{3}d_{4}-80c_{2}c_{6}d_{1}d_{5}-128c_{2}c_{6}d_{2}d_{4}-72c_{2}c_{6}d_{3}{}^{2}\\ \; & -64c_{2}c_{7}d_{1}d_{4}-96c_{2}c_{7}d_{2}d_{3}-48c_{2}c_{8}d_{1}d_{3}-32c_{2}c_{8}d_{2}{}^{2}-32c_{2}c_{9}d_{1}d_{2}-192c_{2}{}^{2}d_{4}d_{6}\\ \; & -100c_{2}{}^{2}d_{5}{}^{2}-128c_{2}c_{3}d_{1}d_{8}-224c_{2}c_{3}d_{2}d_{7}-288c_{2}c_{3}d_{3}d_{6}-320c_{2}c_{3}d_{4}d_{5}\\ \; & -112c_{2}c_{4}d_{1}d_{7}-192c_{2}c_{4}d_{2}d_{6}-240c_{2}c_{4}d_{3}d_{5}-128c_{2}c_{4}d_{4}{}^{2}-96c_{2}c_{5}d_{1}d_{6}\\ \; & -160c_{2}c_{5}d_{2}d_{5}-192c_{2}c_{5}d_{3}d_{4}-80c_{2}c_{6}d_{1}d_{5}-128c_{2}c_{6}d_{2}d_{4}-72c_{2}c_{6}d_{3}{}^{2}-64c_{2}c_{7}d_{1}d_{4}\\ \; & -96c_{2}c_{7}d_{2}d_{3}-48c_{2}c_{8}d_{1}d_{3}-32c_{2}c_{8}d_{2}{}^{2}-32c_{2}c_{9}d_{1}d_{2}-8c_{2}c_{10}d_{1}{}^{2}-56c_{3}{}^{2}d_{1}d_{7}\\ \; & -96c_{3}{}^{2}d_{2}d_{6}-120c_{3}{}^{2}d_{3}d_{5}-64c_{3}{}^{2}d_{4}{}^{2}-96c_{3}c_{4}d_{1}d_{6}-160c_{3}c_{4}d_{2}d_{5}-192c_{3}c_{4}d_{3}d_{4}\\ \; & -80c_{3}c_{5}d_{1}d_{5}-128c_{3}c_{5}d_{2}d_{4}-72c_{3}c_{5}d_{3}{}^{2}-64c_{3}c_{6}d_{1}d_{4}-96c_{3}c_{6}d_{2}d_{3}-48c_{3}c_{7}d_{1}d_{3}\\ \; & -32c_{3}c_{7}d_{2}{}^{2}-32c_{3}c_{8}d_{1}d_{2}-8c_{3}c_{9}d_{1}{}^{2}-40c_{4}{}^{2}d_{1}d_{5}-64c_{4}{}^{2}d_{2}d_{4}-36c_{4}{}^{2}d_{3}{}^{2}\\ \; & -64c_{4}c_{5}d_{1}d_{4}-96c_{4}c_{5}d_{2}d_{3}-48c_{4}c_{6}d_{1}d_{3}-32c_{4}c_{6}d_{2}{}^{2}-32c_{4}c_{7}d_{1}d_{2}-8c_{4}c_{8}d_{1}{}^{2}\\ \; & \left.-24c_{5}{}^{2}d_{1}d_{3}-16c_{5}{}^{2}d_{2}{}^{2}-32c_{5}c_{6}d_{1}d_{2}-8c_{5}c_{7}d_{1}{}^{2}-4c_{6}{}^{2}d_{1}{}^{2}\right) \end{array},$$

$$\begin{array}{cc} \; & d_{14}=-\frac{1}{14d_{1}\left(Q^{2}\beta^{2}-4c_{0}{}^{2}\right)}\left(26Q^{2}\beta^{2}d_{2}d_{13}+36Q^{2}\beta^{2}d_{3}d_{12}+44Q^{2}\beta^{2}d_{4}d_{11}\right.\\ \; & +50Q^{2}\beta^{2}d_{5}d_{10}+54Q^{2}\beta^{2}d_{6}d_{9}+56Q^{2}\beta^{2}d_{7}d_{8}+26Q^{2}\beta d_{1}d_{13}+48Q^{2}\beta d_{2}d_{12}\\ \; & +66Q^{2}\beta d_{3}d_{11}+80Q^{2}\beta d_{4}d_{10}+90Q^{2}\beta d_{5}d_{9}+96Q^{2}\beta d_{6}d_{8}+49Q^{2}\beta d_{7}{}^{2}\\ \; & +12Q^{2}d_{1}d_{12}+22Q^{2}d_{2}d_{11}+30Q^{2}d_{3}d_{10}+36Q^{2}d_{4}d_{9}+40Q^{2}d_{5}d_{8}+42Q^{2}d_{6}d_{7}\\ \; & -104c_{0}{}^{2}d_{2}d_{13}-144c_{0}{}^{2}d_{3}d_{12}-176c_{0}{}^{2}d_{4}d_{11}-200c_{0}{}^{2}d_{5}d_{10}-216c_{0}{}^{2}d_{6}d_{9}\\ \; & -224c_{0}{}^{2}d_{7}d_{8}-104c_{0}c_{1}d_{1}d_{13}-192c_{0}c_{1}d_{2}d_{12}-264c_{0}c_{1}d_{3}d_{11}-320c_{0}c_{1}d_{4}d_{10}\\ \; & -360c_{0}c_{1}d_{5}d_{9}-384c_{0}c_{1}d_{6}d_{8}-196c_{0}c_{1}d_{7}{}^{2}-96c_{0}c_{2}d_{1}d_{12}-176c_{0}c_{2}d_{2}d_{11}\\ \; & -240c_{0}c_{2}d_{3}d_{10}-288c_{0}c_{2}d_{4}d_{9}-320c_{0}c_{2}d_{5}d_{8}-336c_{0}c_{2}d_{6}d_{7}-88c_{0}c_{3}d_{1}d_{11}\\ \; & -160c_{0}c_{3}d_{2}d_{10}-216c_{0}c_{3}d_{3}d_{9}-256c_{0}c_{3}d_{4}d_{8}-280c_{0}c_{3}d_{5}d_{7}-144c_{0}c_{3}d_{6}{}^{2}\\ \; & -80c_{0}c_{4}d_{1}d_{10}-144c_{0}c_{4}d_{2}d_{9}-192c_{0}c_{4}d_{3}d_{8}-224c_{0}c_{4}d_{4}d_{7}-240c_{0}c_{4}d_{5}d_{6}\\ \; & -72c_{0}c_{5}d_{1}d_{9}-128c_{0}c_{5}d_{2}d_{8}-168c_{0}c_{5}d_{3}d_{7}-192c_{0}c_{5}d_{4}d_{6}-100c_{0}c_{5}d_{5}{}^{2}\\ \; & -64c_{0}c_{6}d_{1}d_{8}-112c_{0}c_{6}d_{2}d_{7}-144c_{0}c_{6}d_{3}d_{6}-160c_{0}c_{6}d_{4}d_{5}-56c_{0}c_{7}d_{1}d_{7}\\ \; & -96c_{0}c_{7}d_{2}d_{6}-120c_{0}c_{7}d_{3}d_{5}-64c_{0}c_{7}d_{4}{}^{2}-48c_{0}c_{8}d_{1}d_{6}-80c_{0}c_{8}d_{2}d_{5}\\ \; & -96c_{0}c_{8}d_{3}d_{4}-40c_{0}c_{9}d_{1}d_{5}-64c_{0}c_{9}d_{2}d_{4}-36c_{0}c_{9}d_{3}{}^{2}-32c_{0}c_{10}d_{1}d_{4}\\ \; & -48c_{0}c_{10}d_{2}d_{3}-24c_{0}c_{11}d_{1}d_{3}-16c_{0}c_{11}d_{2}{}^{2}-16c_{0}c_{12}d_{1}d_{2}-4c_{0}c_{13}d_{1}{}^{2}\\ \; & -48c_{1}{}^{2}d_{1}d_{12}-88c_{1}{}^{2}d_{2}d_{11}-120c_{1}{}^{2}d_{3}d_{10}-144c_{1}{}^{2}d_{4}d_{9}-160c_{1}{}^{2}d_{5}d_{8}\\ \; & -168c_{1}{}^{2}d_{6}d_{7}-88c_{1}c_{2}d_{1}d_{11}-160c_{1}c_{2}d_{2}d_{10}-216c_{1}c_{2}d_{3}d_{9}-256c_{1}c_{2}d_{4}d_{8}\\ \; & -280c_{1}c_{2}d_{5}d_{7}-144c_{1}c_{2}d_{6}{}^{2}-80c_{1}c_{3}d_{1}d_{10}-144c_{1}c_{3}d_{2}d_{9}-192c_{1}c_{3}d_{3}d_{8}\\ \; & -224c_{1}c_{3}d_{4}d_{7}-240c_{1}c_{3}d_{5}d_{6}-72c_{1}c_{4}d_{1}d_{9}-128c_{1}c_{4}d_{2}d_{8}-168c_{1}c_{4}d_{3}d_{7}\\ \; & -192c_{1}c_{4}d_{4}d_{6}-100c_{1}c_{4}d_{5}{}^{2}-64c_{1}c_{5}d_{1}d_{8}-112c_{1}c_{5}d_{2}d_{7}-144c_{1}c_{5}d_{3}d_{6}\\ \; & -160c_{1}c_{5}d_{4}d_{5}-56c_{1}c_{6}d_{1}d_{7}-96c_{1}c_{6}d_{2}d_{6}-120c_{1}c_{6}d_{3}d_{5}-64c_{1}c_{6}d_{4}{}^{2}\\ \; & -48c_{1}c_{7}d_{1}d_{6}-80c_{1}c_{7}d_{2}d_{5}-96c_{1}c_{7}d_{3}d_{4}-40c_{1}c_{8}d_{1}d_{5}-64c_{1}c_{8}d_{2}d_{4}-36c_{1}c_{8}d_{3}{}^{2}\\ \; & -32c_{1}c_{9}d_{1}d_{4}-48c_{1}c_{9}d_{2}d_{3}-24c_{1}c_{10}d_{1}d_{3}-16c_{1}c_{10}d_{2}{}^{2}-16c_{1}c_{11}d_{1}d_{2}-4c_{1}c_{12}d_{1}{}^{2}\\ \; & -40c_{2}{}^{2}d_{1}d_{10}-72c_{2}{}^{2}d_{2}d_{9}-96c_{2}{}^{2}d_{3}d_{8}-112c_{2}{}^{2}d_{4}d_{7}-120c_{2}{}^{2}d_{5}d_{6}\\ \; & -72c_{2}c_{3}d_{1}d_{9}-128c_{2}c_{3}d_{2}d_{8}-168c_{2}c_{3}d_{3}d_{7}-192c_{2}c_{3}d_{4}d_{6}-100c_{2}c_{3}d_{5}{}^{2}\\ \; & -64c_{2}c_{4}d_{1}d_{8}-112c_{2}c_{4}d_{2}d_{7}-144c_{2}c_{4}d_{3}d_{6}-160c_{2}c_{4}d_{4}d_{5}-56c_{2}c_{5}d_{1}d_{7}\\ \; & -96c_{2}c_{5}d_{2}d_{6}-120c_{2}c_{5}d_{3}d_{5}-64c_{2}c_{5}d_{4}{}^{2}-48c_{2}c_{6}d_{1}d_{6}-80c_{2}c_{6}d_{2}d_{5}-96c_{2}c_{6}d_{3}d_{4}\\ \; & -40c_{2}c_{7}d_{1}d_{5}-64c_{2}c_{7}d_{2}d_{4}-36c_{2}c_{7}d_{3}{}^{2}-32c_{2}c_{8}d_{1}d_{4}-48c_{2}c_{8}d_{2}d_{3}-24c_{2}c_{9}d_{1}d_{3}\\ \; & -16c_{2}c_{9}d_{2}{}^{2}-16c_{2}c_{10}d_{1}d_{2}-4c_{2}c_{11}d_{1}{}^{2}-32c_{3}{}^{2}d_{1}d_{8}-56c_{3}{}^{2}d_{2}d_{7}-72c_{3}{}^{2}d_{3}d_{6}\\ \; & -80c_{3}{}^{2}d_{4}d_{5}-56c_{3}c_{4}d_{1}d_{7}-96c_{3}c_{4}d_{2}d_{6}-120c_{3}c_{4}d_{3}d_{5}-64c_{3}c_{4}d_{4}{}^{2}-48c_{3}c_{5}d_{1}d_{6}\\ \; & -80c_{3}c_{5}d_{2}d_{5}-96c_{3}c_{5}d_{3}d_{4}-40c_{3}c_{6}d_{1}d_{5}-64c_{3}c_{6}d_{2}d_{4}-36c_{3}c_{6}d_{3}{}^{2}-32c_{3}c_{7}d_{1}d_{4}\\ \; & -48c_{3}c_{7}d_{2}d_{3}-24c_{3}c_{8}d_{1}d_{3}-16c_{3}c_{8}d_{2}{}^{2}-16c_{3}c_{9}d_{1}d_{2}-4c_{3}c_{10}d_{1}{}^{2}-24c_{4}{}^{2}d_{1}d_{6}\\ \; & -40c_{4}{}^{2}d_{2}d_{5}-48c_{4}{}^{2}d_{3}d_{4}-40c_{4}c_{5}d_{1}d_{5}-64c_{4}c_{5}d_{2}d_{4}-36c_{4}c_{5}d_{3}{}^{2}-32c_{4}c_{6}d_{1}d_{4}\\ \; & -48c_{4}c_{6}d_{2}d_{3}-24c_{4}c_{7}d_{1}d_{3}-16c_{4}c_{7}d_{2}{}^{2}-16c_{4}c_{8}d_{1}d_{2}-4c_{4}c_{9}d_{1}{}^{2}-16c_{5}{}^{2}d_{1}d_{4}\\ \; & \left.-24c_{5}{}^{2}d_{2}d_{3}-24c_{5}c_{6}d_{1}d_{3}-16c_{5}c_{6}d_{2}{}^{2}-16c_{5}c_{7}d_{1}d_{2}-4c_{5}c_{8}d_{1}{}^{2}-8c_{6}{}^{2}d_{1}d_{2}-4c_{6}c_{7}d_{1}{}^{2}\right) \end{array},$$

$$\begin{array}{cc} \; & d_{15}=-\frac{1}{30d_{1}\left(Q^{2}\beta^{2}-4c_{0}{}^{2}\right)}\left(56Q^{2}\beta^{2}d_{2}d_{14}+78Q^{2}\beta^{2}d_{3}d_{13}+96Q^{2}\beta^{2}d_{4}d_{12}\right.\\ \; & +110Q^{2}\beta^{2}d_{5}d_{11}+120Q^{2}\beta^{2}d_{6}d_{10}+126Q^{2}\beta^{2}d_{7}d_{9}+64Q^{2}\beta^{2}d_{8}{}^{2}+56Q^{2}\beta d_{1}d_{14}\\ \; & +104Q^{2}\beta d_{2}d_{13}+144Q^{2}\beta d_{3}d_{12}+176Q^{2}\beta d_{4}d_{11}+224Q^{2}\beta d_{7}d_{8}+26Q^{2}d_{1}d_{13}\\ \; & +48Q^{2}d_{2}d_{12}+66Q^{2}d_{3}d_{11}+80Q^{2}d_{4}d_{10}+90Q^{2}d_{5}d_{9}+96Q^{2}d_{6}d_{8}-384c_{0}{}^{2}d_{4}d_{12}\\ \; & -440c_{0}{}^{2}d_{5}d_{11}-224c_{0}c_{1}d_{1}d_{14}-480c_{0}{}^{2}d_{6}d_{10}-504c_{0}{}^{2}d_{7}d_{9}-128c_{0}c_{8}d_{4}{}^{2}\\ \; & -72c_{0}c_{10}d_{3}{}^{2}-32c_{0}c_{12}d_{2}{}^{2}-8c_{0}c_{14}d_{1}{}^{2}-200c_{1}c_{5}d_{5}{}^{2}-128c_{1}c_{7}d_{4}{}^{2}-32c_{1}c_{11}d_{2}{}^{2}\\ \; & -8c_{1}c_{13}d_{1}{}^{2}-200c_{2}c_{4}d_{5}{}^{2}-32c_{2}c_{10}d_{2}{}^{2}-8c_{2}c_{12}d_{1}{}^{2}-72c_{3}{}^{2}d_{1}d_{9}-128c_{3}{}^{2}d_{2}d_{8}\\ \; & -168c_{3}{}^{2}d_{3}d_{7}-192c_{3}{}^{2}d_{4}d_{6}-32c_{3}c_{9}d_{2}{}^{2}-8c_{3}c_{11}d_{1}{}^{2}-120c_{4}{}^{2}d_{3}d_{5}-32c_{4}c_{8}d_{2}{}^{2}\\ \; & -64c_{5}{}^{2}d_{2}d_{4}-32c_{5}c_{7}d_{2}{}^{2}-24c_{6}{}^{2}d_{1}d_{3}-224c_{0}{}^{2}d_{2}d_{14}-312c_{0}{}^{2}d_{3}d_{13}-392c_{0}c_{2}d_{7}{}^{2}\\ \; & -288c_{0}c_{4}d_{6}{}^{2}-200c_{0}c_{6}d_{5}{}^{2}-104c_{1}{}^{2}d_{1}d_{13}-192c_{1}{}^{2}d_{2}d_{12}-264c_{1}{}^{2}d_{3}d_{11}\\ \; & -320c_{1}{}^{2}d_{4}d_{10}-360c_{1}{}^{2}d_{5}d_{9}-384c_{1}{}^{2}d_{6}d_{8}-288c_{1}c_{3}d_{6}{}^{2}-88c_{2}{}^{2}d_{1}d_{11}-160c_{2}{}^{2}d_{2}d_{10}\\ \; & -216c_{2}{}^{2}d_{3}d_{9}-256c_{2}{}^{2}d_{4}d_{8}-280c_{2}{}^{2}d_{5}d_{7}-128c_{2}c_{6}d_{4}{}^{2}-128c_{3}c_{5}d_{4}{}^{2}-56c_{4}{}^{2}d_{1}d_{7}\\ \; & -96c_{4}{}^{2}d_{2}d_{6}-8c_{4}c_{10}d_{1}{}^{2}-8c_{5}c_{9}d_{1}{}^{2}-8c_{6}c_{8}d_{1}{}^{2}-576c_{0}c_{3}d_{4}d_{9}-160c_{0}c_{5}d_{1}d_{10}\\ \; & -288c_{0}c_{5}d_{2}d_{9}-384c_{0}c_{6}d_{4}d_{6}-64c_{0}c_{11}d_{1}d_{4}-160c_{1}c_{8}d_{2}d_{5}-72c_{1}c_{9}d_{3}{}^{2}\\ \; & -384c_{2}c_{3}d_{3}d_{8}-336c_{2}c_{4}d_{3}d_{7}-384c_{2}c_{4}d_{4}d_{6}-80c_{2}c_{8}d_{1}d_{5}-128c_{2}c_{8}d_{2}d_{4}\\ \; & -72c_{2}c_{8}d_{3}{}^{2}-64c_{2}c_{9}d_{1}d_{4}-96c_{2}c_{9}d_{2}d_{3}-72c_{3}c_{7}d_{3}{}^{2}-72c_{4}c_{6}d_{3}{}^{2}-40c_{5}{}^{2}d_{1}d_{5}\\ \; & -96c_{5}c_{6}d_{2}d_{3}-576c_{0}c_{1}d_{3}d_{12}-704c_{0}c_{1}d_{4}d_{11}-800c_{0}c_{1}d_{5}d_{11}-864c_{0}c_{1}d_{6}d_{9}\\ \; & -208c_{0}c_{2}d_{1}d_{13}-528c_{0}c_{2}d_{3}d_{11}-640c_{0}c_{2}d_{4}d_{10}-720c_{0}c_{2}d_{5}d_{9}-768c_{0}c_{2}d_{6}d_{8}\\ \; & -192c_{0}c_{3}d_{1}d_{12}-640c_{0}c_{3}d_{5}d_{8}-320c_{0}c_{4}d_{2}d_{10}-512c_{0}c_{4}d_{4}d_{8}-560c_{0}c_{4}d_{5}d_{7}\\ \; & -448c_{0}c_{5}d_{4}d_{7}-480c_{0}c_{5}d_{5}d_{6}-144c_{0}c_{6}d_{1}d_{9}-256c_{0}c_{6}d_{2}d_{8}\\ \; & -128c_{0}c_{7}d_{1}d_{8}-320c_{0}c_{7}d_{4}d_{5}-192c_{0}c_{9}d_{3}d_{4}-96c_{0}c_{11}d_{2}d_{3}-48c_{0}c_{12}d_{1}d_{3}\\ \; & -32c_{0}c_{13}d_{1}d_{2}-672c_{1}c_{2}d_{6}d_{7}-320c_{1}c_{3}d_{2}d_{10}-432c_{1}c_{3}d_{3}d_{9}-512c_{1}c_{3}d_{4}d_{8}\\ \; & -560c_{1}c_{3}d_{5}d_{7}-160c_{1}c_{4}d_{1}d_{10}-288c_{1}c_{4}d_{2}d_{9}-384c_{1}c_{4}d_{3}d_{8}-448c_{1}c_{4}d_{4}d_{7}\\ \; & -480c_{1}c_{4}d_{5}d_{6}-144c_{1}c_{5}d_{1}d_{9}-256c_{1}c_{5}d_{2}d_{8}-336c_{1}c_{5}d_{3}d_{7}-48c_{1}c_{11}d_{1}d_{3}-32c_{1}c_{12}d_{1}d_{2}\\ \; & -448c_{2}c_{3}d_{4}d_{7}-480c_{2}c_{3}d_{5}d_{6}-144c_{2}c_{4}d_{1}d_{9}-256c_{2}c_{4}d_{2}d_{8}-224c_{2}c_{5}d_{2}d_{7}-160c_{2}c_{7}d_{2}d_{5}\\ \; & -192c_{2}c_{7}d_{3}d_{4}-48c_{2}c_{10}d_{1}d_{3}-32c_{2}c_{11}d_{1}d_{2}-96c_{3}c_{6}d_{1}d_{6}-160c_{3}c_{6}d_{2}d_{5}-48c_{3}c_{9}d_{1}d_{3}\\ \; & -32c_{3}c_{10}d_{1}d_{2}-48c_{4}c_{8}d_{1}d_{3}-32c_{4}c_{9}d_{1}d_{2}-48c_{5}c_{7}d_{1}d_{3}-32c_{5}c_{8}d_{1}d_{2}\\ \; & -416c_{0}c_{1}d_{2}d_{13}-704c_{0}c_{1}d_{4}d_{11}-384c_{0}c_{2}d_{2}d_{12}-720c_{0}c_{2}d_{5}d_{9}-352c_{0}c_{3}d_{2}d_{11}\\ \; & -672c_{0}c_{3}d_{6}d_{7}-176c_{0}c_{4}d_{1}d_{11}-432c_{0}c_{4}d_{3}d_{9}-512c_{0}c_{4}d_{4}d_{8}-560c_{0}c_{4}d_{5}d_{7}\\ \; & -384c_{0}c_{5}d_{3}d_{8}-288c_{0}c_{7}d_{3}d_{6}-112c_{0}c_{8}d_{1}d_{7}-192c_{0}c_{8}d_{2}d_{6}-240c_{0}c_{8}d_{3}d_{5}\\ \; & -96c_{0}c_{9}d_{1}d_{6}-160c_{0}c_{9}d_{2}d_{5}-128c_{0}c_{10}d_{2}d_{4}-192c_{1}c_{2}d_{1}d_{12}-352c_{1}c_{2}d_{2}d_{11}\\ \; & -480c_{1}c_{2}d_{3}d_{10}-576c_{1}c_{2}d_{4}d_{9}-640c_{1}c_{2}d_{5}d_{8}-176c_{1}c_{3}d_{1}d_{11}-480c_{1}c_{4}d_{5}d_{6}\\ \; & -384c_{1}c_{5}d_{4}d_{6}-128c_{1}c_{6}d_{1}d_{8}-224c_{1}c_{6}d_{2}d_{7}-288c_{1}c_{6}d_{3}d_{6}-320c_{1}c_{6}d_{4}d_{5}\\ \; & -112c_{1}c_{7}d_{1}d_{7}-192c_{1}c_{7}d_{2}d_{6}-240c_{1}c_{7}d_{3}d_{5}-192c_{1}c_{8}d_{3}d_{4}-64c_{1}c_{10}d_{1}d_{4}\\ \; & -96c_{1}c_{10}d_{2}d_{3}-160c_{2}c_{3}d_{1}d_{10}-288c_{2}c_{3}d_{2}d_{9}-288c_{2}c_{5}d_{3}d_{6}-320c_{2}c_{5}d_{4}d_{5}\\ \; & -112c_{2}c_{6}d_{1}d_{7}-192c_{2}c_{6}d_{2}d_{6}-240c_{2}c_{6}d_{3}d_{5}-96c_{2}c_{7}d_{1}d_{6}-128c_{3}c_{4}d_{1}d_{8}\\ \; & -224c_{2}c_{4}d_{2}d_{7}-320c_{3}c_{4}d_{4}d_{5}-112c_{3}c_{5}d_{1}d_{7}-192c_{3}c_{5}d_{2}d_{6}-240c_{3}c_{5}d_{3}d_{5}\\ \; & -96c_{3}c_{6}d_{1}d_{6}-160c_{3}c_{6}d_{2}d_{5}-192c_{3}c_{6}d_{3}d_{4}-80c_{3}c_{7}d_{1}d_{5}-128c_{3}c_{7}d_{2}d_{4}\\ \; & -64c_{3}c_{8}d_{1}d_{4}-96c_{3}c_{8}d_{2}d_{3}-96c_{4}c_{5}d_{1}d_{6}-160c_{4}c_{5}d_{2}d_{5}-80c_{4}c_{6}d_{1}d_{5}\\ \; & -128c_{4}c_{6}d_{2}d_{4}-64c_{4}c_{7}d_{1}d_{4}-32c_{6}c_{7}d_{1}d_{2}+200Q^{2}\beta d_{5}d_{10}+216Q^{2}\beta d_{6}d_{9}\\ \; & +49Q^{2}d_{7}{}^{2}-256c_{0}{}^{2}d_{8}{}^{2}-896c_{1}d_{7}d_{8}-480c_{0}c_{3}d_{3}d_{10}-336c_{0}c_{6}d_{3}d_{7}\\ \; & -224c_{0}c_{7}d_{2}d_{7}-80c_{0}c_{10}d_{1}d_{5}-196c_{1}{}^{2}d_{7}{}^{2}-96c_{1}c_{8}d_{1}d_{6}-80c_{1}c_{9}d_{1}d_{5}\\ \; & -128c_{1}c_{9}d_{2}d_{4}-144c_{2}{}^{2}d_{6}{}^{2}-128c_{2}c_{5}d_{1}d_{8}-100c_{3}{}^{2}d_{5}{}^{2}-288c_{3}c_{4}d_{3}d_{6}\\ \; & -64c_{4}{}^{2}d_{4}{}^{2}-192c_{4}c_{5}d_{3}d_{4}-96c_{4}c_{7}d_{2}d_{3}-36c_{5}{}^{2}d_{3}{}^{2}-64c_{5}d_{1}d_{4}-16c_{6}{}^{2}d_{2}{}^{2}\\ \; & \left.-4c_{7}{}^{2}d_{1}{}^{2}\right) \end{array}.$$
